# The class II myosin MYH4 safeguards genome integrity and suppresses tumor progression

**DOI:** 10.1172/JCI188165

**Published:** 2025-06-02

**Authors:** Jayashree Thatte, Ana Moisés da Silva, Judit Börcsök, Thorkell Gudjónsson, Jan Benada, Xin Li, Muthiah Bose, Hanneke van der Gulden, Ji-Ying Song, Renée Menezes, Elena Martín-Doncel, Luis Toledo, Valdemaras Petrosius, Cord Brakebusch, Jos Jonkers, Finn Cilius Nielsen, Maria Rossing, Claus S. Sørensen

**Affiliations:** 1 Biotech Research and Innovation Center (BRIC), Faculty of Medical and Health Sciences, University of Copenhagen, Copenhagen, Denmark.; 2 Division of Molecular Pathology, The Netherlands Cancer Institute, Amsterdam, Netherlands.; 3 Oncode Institute, Utrecht, Netherlands.; 4 Center for Genomic Medicine, Rigshospitalet, Copenhagen University Hospital, Copenhagen, Denmark.; 5 Experimental Animal Pathology and; 6 Biostatistics Centre & Division of Psychosocial Research and Epidemiology, The Netherlands Cancer Institute, Amsterdam, Netherlands.; 7 Center for Chromosome Stability, Institute for Cellular and Molecular Medicine, Faculty of Health and Medical Sciences, University of Copenhagen, Copenhagen, Denmark.; 8 Department of Clinical Medicine, University of Copenhagen, Copenhagen, Denmark.

**Keywords:** Cell biology, Clinical Research, Oncology, Breast cancer, Genetic instability, Tumor suppressors

## Abstract

Loss-of-function mutations in genome maintenance genes fuel tumorigenesis through increased genomic instability. A subset of these tumor suppressors are challenging to identify due to context dependency, including functional interactions with other genes and pathways. Here, we searched for potential causal genes that impact tumor development and/or progression in breast cancer through functional-genetic screening of candidate genes. *MYH4*, encoding a class II myosin, emerged as a top hit impacting genomic stability. We show that MYH4 suppresses DNA replication stress by promoting replication licensing and replication fork progression. Moreover, we observed a strong synergistic relationship among class II myosins in suppressing replication-associated DNA damage. Genomic analysis of Pan-Cancer Analysis of Whole Genomes project breast cancer samples revealed frequent concomitant loss of *TP53* with *MYH4* and class II myosins on chromosome 17p. Notably, *Myh4* disruption accelerated mouse mammary tumorigenesis in a *Trp53*-deficient background. In conclusion, our results suggest an unanticipated function of MYH4 in p53-mediated tumor suppression that can explain their combined loss in breast cancer.

## Abstract 2

This study identifies tumor suppressive role of MYH4, a gene frequently co-lost with TP53. MYH4 suppresses replication stress and its deficiency is a potential vulnerability towards WEE1 inhibitors.

## Graphical abstract

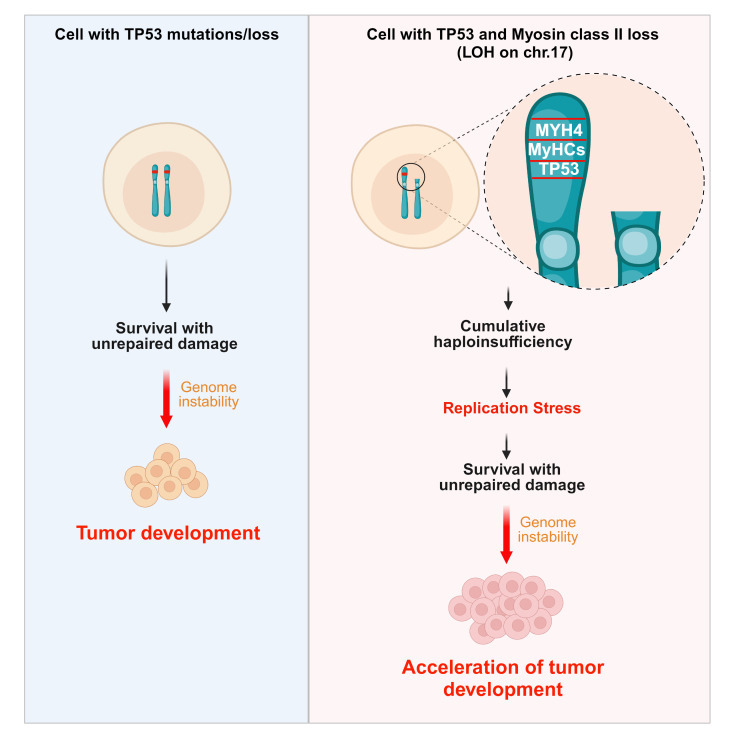

## Introduction

Dysfunctional genome maintenance frequently drives tumor development (1). Many breast cancer tumor suppressors are genome maintenance genes, such as *BRCA1, BRCA2, ATM*, and *TP53* (2–5). Given the clinical importance, it is crucial to identify new causal genes and processes, including the evaluation of their influence on cancer development and progression (6). Multiple factors and pathways involved in replication and DNA repair have emerged in unbiased functional studies, including those involved in cytoskeletal remodeling and various aspects of cancer development (7–9).

Recent findings have suggested a role for nuclear F-actin and actin-related proteins, including myosins, in genome maintenance. Myosins represent a varied superfamily of molecular motors that, through their interaction with actin, convert the energy released from ATP hydrolysis into mechanical force. Notably, in *Drosophila* and mouse cells, an actin-myosin–dependent mechanism facilitates the movement of heterochromatic double-strand breaks for homologous recombination repair (10). Actin and class II myosins are also suggested to mediate the mobility and repair of broken replication forks following prolonged fork stalling (11). Moreover, it has been demonstrated that motor myosin VI plays a role in protecting stalled forks from nucleolytic degradation (12). Growing evidence indicates that myosins impact cytokinesis failure, chromosomal and centrosomal amplification, as well as multipolar spindle formation in cancer cells (13, 14). The functions of myosins in the process of tumor development have become evident only in the last few years. Myosins have attracted attention in the context of cancer therapy due to their impact on genetic and chromosomal instability (15). Additionally, metastasis in cancer is achieved by acto-myosin contraction and stress fiber formation (15–19). Notably, *MYO1C* has been reported as a tumor suppressor gene that is often concomitantly lost with *TP53* on chromosome 17p (20). Expression analysis and genetic screening showed downregulation of *Myo1c* in rat endometrial carcinomas, suggesting potential haploinsufficiency of this gene in tumor suppression (21).

A comprehensive study from Scott Lowe’s lab used an RNAi approach to suggest the involvement of additional genes at the *Trp53* locus in mouse lymphomagenesis (22). Their experiments revealed 17 additional genes, including several class II myosins such as *MYH1*, *MYH3*, and *MYH8*, whose depletion promoted tumorigenesis. These results imply that the impact of 17p deletion on tumorigenesis may not only be due to p53 loss, and such segmental deletion events can influence tumor progression and resistance to treatment by disrupting multiple genes and associated mechanisms. Overall, in each specific cancer type, myosins may play distinct yet important roles throughout the tumorigenic process. Unraveling the specific functions of myosins and understanding their associated mechanisms is therefore relevant for understanding cancer biology and it may fuel future cancer therapy strategies. In this study, we identified myosin heavy chain 4 (MYH4), a class II myosin family protein, as a genome maintenance factor that suppresses replication stress. Moreover, our in vivo findings indicate *Myh4* as a chromosome 17p gene involved in mammary tumor progression. Altogether, our data highlight how MYH4 can suppress breast cancer and indicate how it guards against replication-associated vulnerabilities, offering significant potential for therapeutic exploitation in targeting these cancers.

## Results

### MYH4 supports genomic integrity.

**Figure 1:**
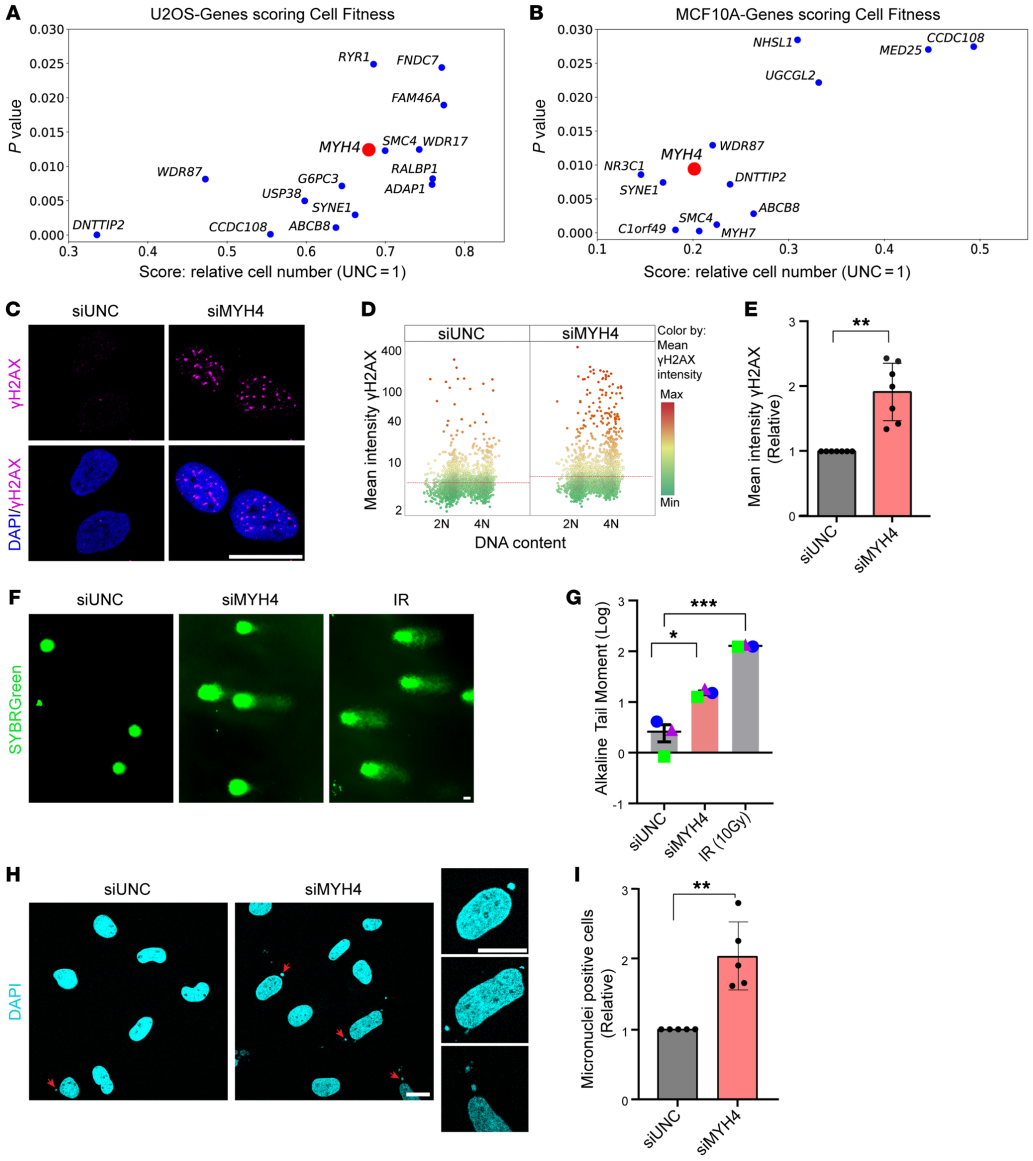
MYH4 supports genomic integrity. (**A** and **B**) Scatter plots depicting genes that scored with 2 or more siRNAs for cell fitness phenotype in U2OS and MCF10A breast cell lines, respectively. MYH4 is highlighted in red. The *y* axis shows the *P* values calculated from the mean of 3 replicates; the *x* axis shows scores for relative cell number, normalized to siUNC = 1. (**C**) U2OS cells transfected with siUNC or siMYH4 (no. 3) for 72 hours and subjected to immunofluorescence analysis to assess γH2AX levels. Representative confocal images are shown; DAPI was used to stain nuclei. Scale bar: 20 μm. (**D**) QIBC plot of U2OS cells transfected with indicated siRNAs and stained for γH2AX. Plot shows mean intensity of γH2AX per nucleus on the *y* axis and total DNA content on the *x* axis. Color legend: green, minimum; yellow, average; red, maximum. (**E**) A bar chart showing relative γH2AX mean intensity of U2OS cells transfected with siMYH4 (no. 3). Data points show 7 biological replicates. Data presented as mean ± SD. (**F**) Representative images of alkaline comet assay from 3 biological replicates. Ionizing radiation–treated (IR-treated) cells were used as a positive control. Scale bar: 20 μm. (**G**) Quantification of comet tail moment from 3 biological replicates. Error bars indicate ±SEM. (**H**) Confocal images showing micronuclei formation (red arrows) after MYH4 knockdown; DAPI was used to stain nuclei. Scale bar: 20 μm. (**I**) Quantification of cells harboring micronuclei; relative values from 5 biological replicates are shown. Data presented as mean ± SD. *P < 0.05; **P < 0.001; ***P < 0.001 by 2-tailed, paired *t* test.

To explore new tumor suppressive factors in breast cancer, we turned to unanticipated genes mutated in early-onset breast cancers as a starting point. There is substantial missing heritability in hereditary breast cancer (HBC), which may be due to a combination of genetic variants in multiple genes. Such genes and variants may not reach thresholds required to be nominated as tumor suppressor genes in HBC, but they may well contribute to both HBC and sporadic breast cancer (6). Thus, we set out to identify rare germline genetic variants in a cohort of patients with early-onset breast cancer (*BRCA1*/*BRCA2*/*TP53* WT) (23). We filtered for genes that harbored a combination of a minimum of 1 stop/start-loss variant and 1 missense variant. This led to a shortlist of 150 genes that were enrolled for siRNA-based screens in U2OS and MCF10A cells. We generated a targeted siRNA library comprising 3 siRNAs designed for each gene. The functional screen was performed to score cell fitness phenotype, as it is often linked to genomic instability as well as oncogenic drivers (Supplemental Figure 1A; supplemental material available online with this article; https://doi.org/10.1172/JCI188165DS1) (24, 25). Interestingly, MYH4 scored highly in both cell lines (Figure 1, A and B). MYH4 belongs to the class II family of muscle proteins and plays a role in cell contractility (26). We enrolled MYH4 for further validation based on the following reasons: (a) the *MYH4* gene is situated on chromosome 17p, a region commonly lost in multiple cancer types (27, 28); (b) the role of MYH4 in genome maintenance and tumorigenesis is not yet explored; and (c) we speculated that noncanonical functions of MYH4 might operate in DNA repair and replication given the newly discovered relevance of actin and myosin in this context (8, 9, 29–32). To further explore an involvement of MYH4 in genome maintenance, we examined γH2AX foci formation as a pseudomarker of DNA damage in U2OS cells. This particular cell line was selected as model because it is well characterized in genome maintenance and cell cycle studies, and it is excellent for quantitative immunofluorescence microscopy techniques. siRNA-mediated depletion of MYH4 (72 hours) caused elevated γH2AX signals, as evidenced by an increase in the mean γH2AX intensity in the nucleus, compared with universal negative control (siUNC; Figure 1, C–E). We repeated these results using multiple siRNAs targeting MYH4 (Supplemental Figure 1B), where 2 out of 3 siRNAs showed significant increases in γH2AX-positive cells. All tested siRNAs exhibited effective targeting of both endogenous MYH4 and exogenous GFP-tagged MYH4, thus validating the reagents used in this study (Supplemental Figure 1, C and D). siRNA no. 3 was chosen for further validations. The observed γH2AX phenotype was consistent across breast cancer cell lines such as MCF-7 (Supplemental Figure 1, E–G). Consistent with elevated γH2AX levels, the alkaline comet assay revealed an increased tail moment for cells transfected with siMYH4, compared with siUNC-transfected cells, indicating the occurrence of DNA breaks upon depletion of MYH4 (Figure 1, F and G). Furthermore, we also detected an increased percentage of cells with micronuclei following MYH4 depletion (Figure 1, H and I). Collectively, these findings suggest that MYH4 plays a role in genome maintenance.

### MYH4 complementation prevents DNA damage accumulation.

**Figure 2:**
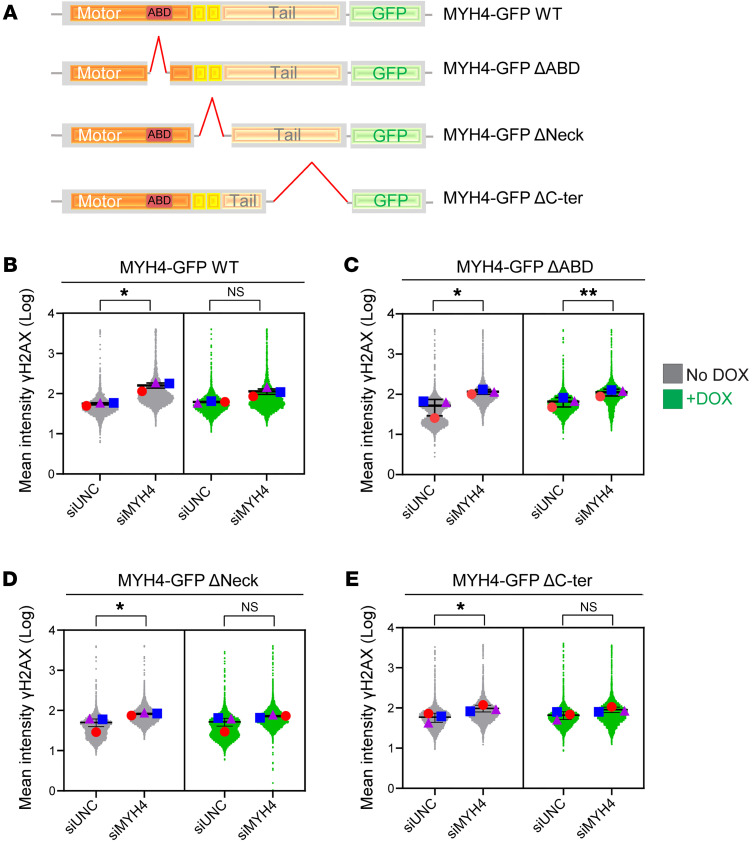
MYH4 complementation prevents DNA damage accumulation. (**A**) A schematic illustration of WT MYH4-GFP and various domain mutants. (**B**–**E**) U2OS cells stably expressing DOX-inducible siMYH4-resistant WT MYH4-GFP or indicated mutants. The cells were transfected with either siUNC or siMYH4 for 48 hours. DOX (1 μg/mL) was added 5 hours after transfection. Density plots (>1000 cells) illustrate the mean intensity of γH2AX across indicated WT or mutant cell lines. Error bars indicate ±SEM. *P < 0.05, **P < 0.001 by 2-tailed, paired *t* test. NS, not significant.

To establish an isogenic system for our experiments, we introduced doxycycline-inducible (DOX-inducible), siRNA-resistant, GFP-tagged MYH4 into U2OS cells using a lentivirus system. Subsequently, we depleted endogenous MYH4 using siMYH4 for 48 hours (Supplemental Figure 2A). In line with prior observations (33), we noted a predominant cytoplasmic localization of MYH4 in the majority of cells and only a subset of cells with minimal nuclear expression (<1%) (Supplemental Figure 2B). Next, we generated several MYH4 mutants lacking different functional domains to examine their phenotypic significance. The schematic representation of MYH4 domains and corresponding mutants is presented in Figure 2A. Different domain mutants that we generated were as follows: (a) actin-binding domain deletion (ΔABD), (b) light chain–binding neck region deletion (ΔNeck), and (c) C-terminal coiled-coil domain deletion (ΔC-ter) (Supplemental Figure 2C). Supplemental Figure 2, D and E show the expression of respective MYH4 mutants in stable cell lines.

Importantly, complementation with WT MYH4 rescued the genomic instability phenotype resulting from MYH4 loss (Figure 2B). Interestingly, expression of the ΔABD mutant lacking the ABD that is highly conserved across the myosin II family did not reverse the phenotype, indicating a role for this domain in maintaining genomic stability (Figure 2C). In contrast, the other 2 mutants exhibited restoration of the phenotype (Figure 2, D and E).

### MYH4 ensures replication licensing and promotes faithful replication.

**Figure 3:**
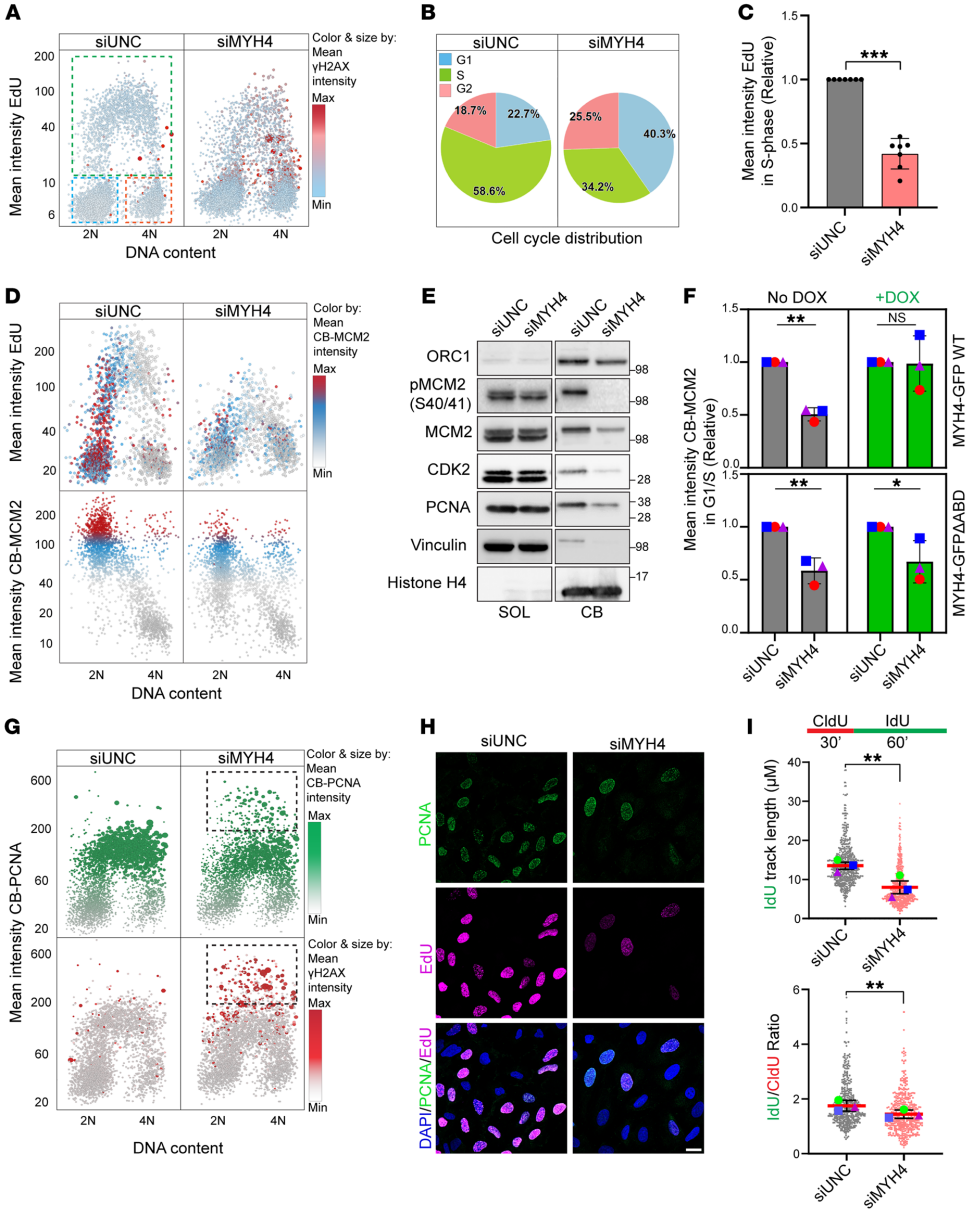
MYH4 ensures replication licensing and promotes faithful replication. (**A**) QIBC plot indicating EdU incorporation of U2OS cells transfected with indicated siRNAs for 48 hours. Boxes indicate cell cycle phases: blue, G_1_; green, S; and red, G_2_. Color threshold indicates mean γH2AX intensity in different cell cycle phases. Blue, minimum; red, maximum. (**B**) A pie chart indicating EdU- and DAPI-based cell cycle distribution of cells. (**C**) Statistics for mean EdU intensity in S phase. Data presented as mean ± SD. ***P < 0.001 by 2-tailed, paired *t* test. (**D**) QIBC plot of cells transfected with the indicated siRNAs and stained for chromatin-bound (CB) MCM2. Top panels: MCM2 distribution in cell cycle phases. Bottom panels: Mean intensity of CB MCM2. Color threshold: gray, minimum; blue, average; red, maximum. (**E**) A Western blot analysis of indicated proteins in soluble (SOL) and CB extracts from cells transfected with indicated siRNAs, *n =* 3. Note: Some of the blots were stripped and reblotted with different antibodies. (**F**) U2OS cells stably expressing DOX inducible, siRNA-resistant MYH4-GFP WT or ΔABD mutant, transfected with indicated siRNAs for 48 hours. The bar plots illustrate relative mean intensity of CB MCM2. Data presented as mean ± SD, *n =* 3. *P < 0.05, **P < 0.001 by 1-way ANOVA with Šidák’s multiple-comparison test. NS, not significant. (**G**) QIBC of cells transfected with the indicated siRNAs and stained for CB-PCNA. Top panel: PCNA distribution in cell cycle phases. Bottom panel: Mean intensity of γH2AX in the same set of cells, indicated with a box. (**H**) Representative confocal images showing PCNA and EdU staining after 48 hours of indicated siRNA transfection (*n =* 3). Scale bar: 20 μm. (**I**) Quantification of DNA fibers from cells transfected with indicated siRNAs and labeled with indicated analogs for indicated time. Error bars indicate ±SEM, *n =* 3. **P < 0.001 by 2-tailed, paired *t* test.

With the understanding that MYH4 loss triggers genomic instability, our subsequent focus was to investigate underlying causes or consequences. We therefore used quantitative image-based cytometry (QIBC) to conduct a quantitative analysis of the G_1_-S-G_2_/M transition at the single-cell level. We observed a significant decrease in the S phase and increased percentage of cells in G_1_ phase when MYH4 was depleted, as analyzed with mean EdU intensity and total intensity DAPI (Figure 3, A–C).

We hypothesized that key processes related to DNA replication are mediated by MYH4. To this end, we first examined whether cell cycle arrest at the G_1_ phase was a consequence of inadequate replication licensing (34). We monitored the presence of the preinitiation complex factor, MCM2 (35, 36), on chromatin using QIBC. Our findings revealed a notable deficiency in MCM2 loading during G_1_ and early-S phase when MYH4 was depleted, as depicted by the blue (average) and red (maximum) signal in the QIBC plot (Figure 3D). This phenotype was consistent across normal breast epithelial cell lines, including WT and *TP53*-knockout (*TP53*-KO) MCF10A cells (Supplemental Figure 3A). To investigate whether DNA damage is the cause or a consequence of this particular phenotype, we conducted kinetics studies. The MCM2 phenotype became apparent 30 hours after siRNA transfection, whereas the γH2AX signal appeared after 48 hours (Supplemental Figure 3B). This pattern suggests that the DNA damage signaling and cell cycle arrest are exacerbated by insufficient replication licensing. Additionally, we observed a significant decrease in the phosphorylation of MCM2 at serines 40 and 41 in MYH4-depleted cells, which is a known marker for initiation of replication (37, 38) (Supplemental Figure 3, C and D). Furthermore, we analyzed the chromatin binding of pre-replication factors (comprising ORC1, CDC6, MCM2, and CDT1) and replication initiation and elongation factors (including CDK2 and PCNA) through Western blotting (35). Depletion of MYH4 led to a notable reduction in both pre-replication and pre-initiation complex components within the chromatin-bound fractions (Figure 3E and Supplemental Figure 3E). Notably, complementation of WT but not the ΔABD form of MYH4 rescued the MCM2 loading (Figure 3F). In summary, these findings show that MYH4 promotes replication origin licensing and proper cell cycle progression.

To further explore the impact of MYH4 on DNA replication, we assayed the PCNA profile of cells, a marker for ongoing DNA replication (39). Control cells exhibited a positive PCNA signal in the S phase, visualized in green, representing replicating cells (Figure 3, G and H). In contrast, MYH4-depleted cells displayed 2 distinct patterns. While the majority of these cells showed reduced chromatin-bound PCNA, a small fraction of MYH4-depleted cells showed increased intensity of PCNA staining. We investigated this further by simultaneously measuring PCNA and γH2AX signals, revealing a strong correlation between high PCNA intensity and elevated levels of DNA damage (Figure 3G and Supplemental Figure 3F). Thus, the high-intensity PCNA foci and their colocalization with γH2AX suggest perturbations at replication forks (40).

Deficiency in replication licensing challenges the timely completion of the replication program and may lead to DNA replication stress. We therefore investigated the replication stress response via ATR kinase activation (41–43) by measuring levels of chromatin-associated phosphorylated RPA (S33), which is an ATR target (44). MYH4 knockdown resulted in a notable accumulation of phosphorylated RPA on chromatin, indicating the stalling of replication forks that are susceptible to collapsing into double-strand breaks. To explore this further, we exposed cells to hydroxyurea (HU), a known replication-stalling agent. As anticipated, we observed an increase in phosphorylated RPA (S33) (Supplemental Figure 3G, right panel) in cells treated with siMYH4 and HU, suggesting that MYH4 plays a role in preventing replication stress.

It has been demonstrated that replication stress leads to the appearance of under-replicated DNA that often manifests via 53BP1 nuclear bodies in the G_1_ phase (45). Consistent with this, we observed elevated levels of 53BP1 bodies in the G_1_ phase following MYH4 knockdown (Supplemental Figure 3H). Moreover, replication stress can be experimentally detected as reduced fork progression. Thus, we monitored DNA replication via DNA fiber assays, where nascent DNA is labeled sequentially with thymidine analogs, 5-chloro-2′-deoxyuridine (CldU) and 5-iodo-2′-deoxyuridine (IdU) (Figure 3I). Knockdown of MYH4 led to a decrease in the overall DNA replication speed, as determined by the length of the IdU-labeled tracks and reduced IdU/CldU ratio, indicating the requirement of MYH4 for efficient DNA replication (Figure 3I and Supplemental Figure 3I). Next, we analyzed various fork types in our fiber assays and found an increased frequency of new origin firing following MYH4 knockdown, potentially linked to perturbations at replication forks. However, under mild replication stress induced by 0.5 mM HU for 1 hour, there was a slight decrease in new origin firing compared with siUNC (Supplemental Figure 3J). Based on our earlier findings, this phenotype is likely attributable to reduced replication licensing, limiting the cells’ ability to respond effectively to replication stress. These findings led us to hypothesize that MYH4-depleted cells will be vulnerable to emerging therapy-relevant drugs such as WEE1 kinase inhibitors that further enhance replication stress and prematurely advance cell cycle progression (46, 47). To test this, we treated both U2OS and *TP53*-KO MCF10A cell lines with varying concentrations of WEE1 inhibitor for 18 hours. We did not proceed to analyze the WT MCF10A cell line, as MYH4 depletion disturbed cell cycle progression in this cell line (Supplemental Figure 3A). As demonstrated in Supplemental Figure 3, K and L, combined MYH4 depletion and WEE1 inhibition led to a noticeable increase in γH2AX levels. Next, we assessed cell fitness in U2OS cells treated with WEE1 inhibitor, in the presence or absence of MYH4. As illustrated in Supplemental Figure 3M, a significant reduction in cell viability was observed when MYH4 was depleted in combination with WEE1 inhibition. Collectively, these data support the notion that MYH4 acts as a protective mechanism against both replication stress and genomic instability.

### Class II myosins exhibit codependency for cell fitness and suppress replication-associated DNA damage.

**Figure 4:**
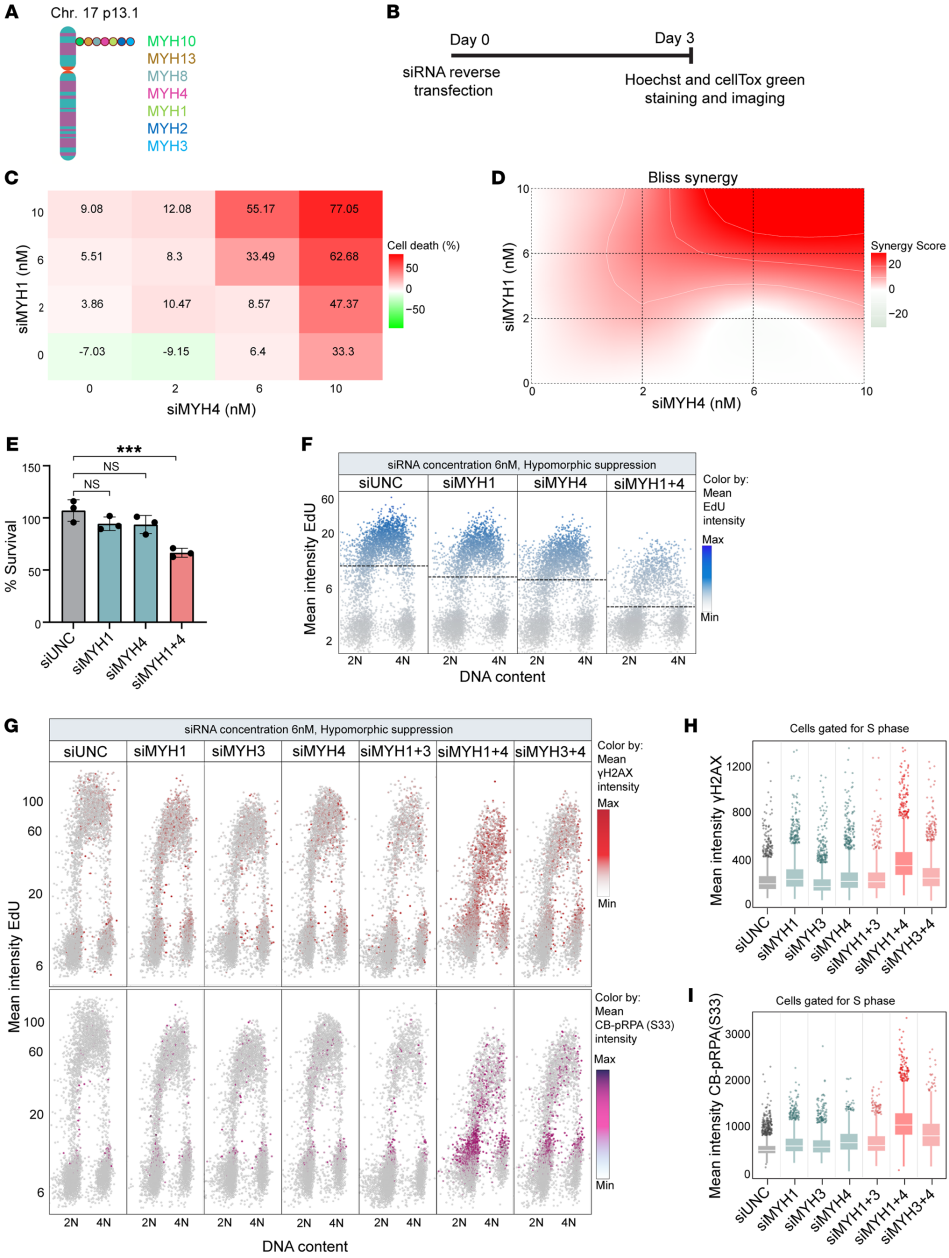
Class II myosins exhibit codependency and suppress replication-associated DNA damage. (**A**) Diagram illustrating chromosome 17 and the genes located in the vicinity of the *MYH4* locus. (**B**) A scheme depicting experimental design and timeline. (**C**) A representative matrix depicting the dose-response relationship for cell viability in U2OS cells following transfection with specified siRNA pairs for a duration of 72 hours, with various siRNA concentrations. The values represent the mean of 3 replicates, normalized to siUNC and calculated as a percentage of cell death. (**D**) The Bliss synergy map corresponding to data in panel **B** is presented. (**E**) A bar chart showing percentage survival of cells transfected with indicated siRNAs at 6 nM concentration each, for 72 hours (related to **C**). Data presented as mean ± SD, *n =* 3. ***P < 0.001 by 1-way ANOVA with Šidák’s multiple-comparison test. NS, not significant. (**F**) QIBC plot indicating EdU incorporation of U2OS cells transfected with the indicated siRNAs (6 nM each) for 48 hours. Dashed line: Average EdU intensity. (**G**) QIBC plot of cells transfected with the indicated siRNAs (6 nM each) for 48 hours. Top panel: The distribution of γH2AX. γH2AX color threshold: gray, minimum; red, average; maroon, maximum. Bottom panel: The distribution of chromatin-bound (CB) p-RPA (S33) in different cell cycle phases. CB p-RPA (S33) color threshold: gray, minimum; magenta, average; purple, maximum. (**H**) Box-and-whisker plot (related to **G**, top panel) showing mean intensity of γH2AX in S phase of cells transfected with indicated siRNAs (6 nM each) for 48 hours. (**I**) Box-and-whisker plot (related to **G**, bottom panel) showing mean intensity of CB p-RPA (S33) in S phase of U2OS cells transfected with indicated siRNAs (6 nM each) for 48 hours. In **H** and **I**, middle lines indicate medians, thick boxes indicate lower (25th, Q1) and upper (75th, Q3) quartiles, and whiskers are at 10th and 90th percentiles. The data points are from a single experiment, representative of 3 independent experiments.

To further explore potential vulnerabilities related to MYH4 deficiency, we noticed that the Cancer Dependency Map (DepMap) indicates a cancer cell fitness codependency relationship between *MYH1*, *MYH3*, and *MYH4* (https://depmap.org/portal/gene/MYH4?tab=overview). These 3 genes all belong to the class II myosins that share a high degree of structural and functional similarity (48), and some of the class II myosins locate to the same locus on chromosome 17p (Figure 4A) (49). Moreover, mass spectrometry analysis of MYH4-GFP interactions indicated its coimmunoprecipitation with MYH1 and MYH3, implying potential functional interactions between these proteins (Supplemental Figure 4A). These observations led us to experimentally probe mutual dependency among different class II myosins. To explore the potential synergistic effect of targeting multiple class II myosins in combination with MYH4, we performed siRNA-based dose-response matrix screening for cell viability by transfecting cells for 72 hours with different concentrations of siRNAs targeting MYH4 and class II myosins alone, or in combination (Figure 4B). Figure 4C and Supplemental Figure 4B depict a representative experiment with siRNAs targeting MYH1 and MYH4, showing that combined inhibition was more effective in killing cells compared with individual siRNA knockdowns. We utilized the Bliss synergy model (SynergyFinder) to assess the synergy score, which unveiled a robust synergistic relationship between MYH4 and the class II myosins. Figure 4D displays a representative synergy map between MYH1 and MYH4, highlighting significant synergy between the respective siRNAs in triggering cell death, particularly at concentrations exceeding 6 nM (Figure 4E). The majority of examined class II myosins exhibited codependency and high Bliss synergy with MYH4, indicated by synergy scores exceeding 10 (Supplemental Figure 4, C–J).

We next investigated whether the observed synergistic effect on cell survival could be attributed to replication-associated DNA damage. We observed that partial depletion (using 6 nM of each siRNA for 48 hours) of MYH1 or MYH4 individually had little impact on replication, as assessed by EdU incorporation assay (Supplemental Figure 4, K and L). However, when these depletions were combined, there was a notable reduction in replication (Figure 4F). This phenotype was also consistent in the *TP53*-KO MCF10A cell line (Supplemental Figure 4M). This finding was further confirmed by assessing the effect of MYH1/MYH3 along with MYH4 on genomic stability. As depicted in Figure 4, G–I, an increase in γH2AX levels and hyperphosphorylation of RPA at S33 was noted when MYH4 was depleted together with either MYH1 or MYH3 in U2OS cells and an increase in γH2AX levels in *TP53*-KO MCF10A cells (Supplemental Figure 4N). The synergistic effect on cell survival observed may thus be linked to the compromised DNA replication processes.

Given the genomic proximity to *TP53* on chromosome 17p, we then investigated the genetic relationship between *TP53* and *MYH4*. As shown in Supplemental Figure 4O, the *TP53*-KO MCF10A cell line combined with siMYH4 exhibited somewhat higher levels of γH2AX compared with WT MCF10A cells, suggesting potential genetic interactions between *TP53* and *MYH4*.

### MYH4 loss accelerates mammary tumorigenesis in a TP53-deficient background.

**Figure 5:**
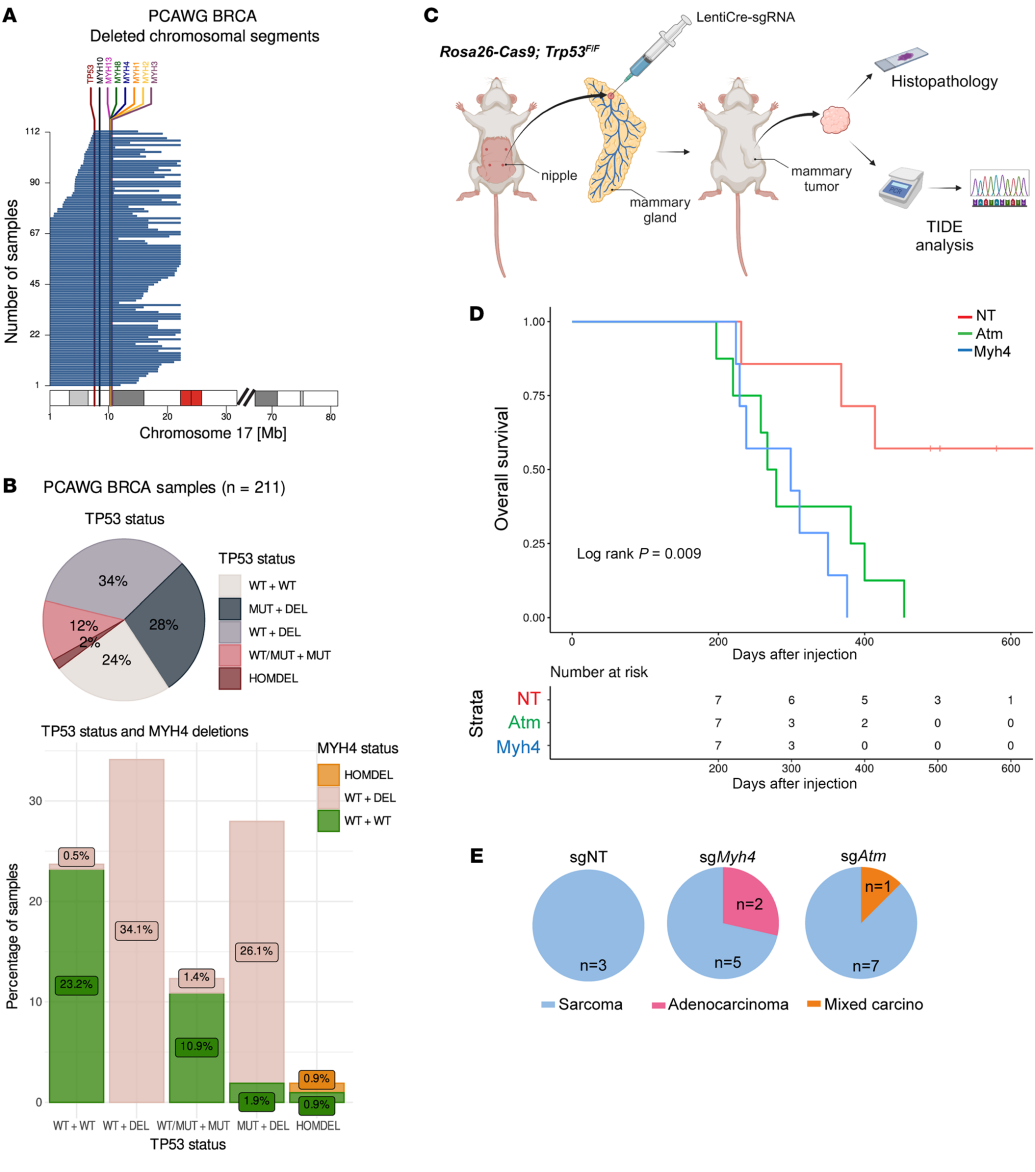
MYH4 loss accelerates mammary tumorigenesis in a *TP53*-deficient background. (**A**) Deleted chromosomal segments that include both *TP53* and *MYH4* in the breast cancer (BRCA) subset of the PCAWG cohort (*n =* 211). *TP53*, *MYH4*, and other myosin class II family genes are highlighted in different colors. (**B**) Top panel: A pie chart depicting *TP53* somatic mutation status of samples in the BRCA subset of the PCAWG cohort, color coded by *TP53* status. Bottom panel: Combined assessment of *TP53* and *MYH4* status of samples in the BRCA subset of the PCAWG cohort, color coded by *MYH4* status. (**C**) Schematic representation of intraductal injection of high-titer lentiviruses encoding Cre and nontargeting sgRNA (sgNT), or sgRNAs targeting *Myh4* or *Atm* alleles in *Rosa26*-*Cas9*; *Trp53^fl/fl^* females. After harvesting, tumor material underwent histopathology and TIDE analysis. (**D**) Kaplan-Meier curves showing overall survival of *Rosa26*-*Cas9*; *Trp53^fl/fl^* mice when injected with sgNT (*n =* 7), sg*Atm* (*n =* 8), or sg*Myh4* (*n =* 7). Mice injected with sg*Atm* and sg*Myh4* show a significant difference when compared with sgNT (*P =* 0.009, log-rank test). Number of animals at risk over time is represented in the table at the bottom. One mouse in the *Atm* group is not represented because it was euthanized due a tumor at 197 days after injection. (**E**) Histopathological classification of the main tumor of each mouse injected with sgNT (*n =* 3), sg*Myh4* (*n =* 7), or sg*Atm* (*n =* 8). The *Atm* group includes a subgroup, mixed carcinoma, where different carcinomas were found in the same lesion. In one specific case, a mix of adenocarcinoma and squamous cell carcinoma was observed.

Considering the interdependence among different class II myosins and their proximity to *TP53* on chromosome 17p, we conducted genomic analysis of the nature and extent of chromosome 17p deletions in breast cancer (BRCA) samples from the Pan-Cancer Analysis of Whole Genomes (PCAWG) project (50). Examination of genomic data from 221 BRCA tumors substantiated earlier findings (51), revealing that approximately half of the tumors exhibited segmental or complete deletion of the 17p arm, encompassing *TP53*, *MYH4*, and other class II myosins (Figure 5A). Of note, this genomic region has several protein-coding genes, including known or putative tumor suppressors.

We further categorized BRCA tumors based on whether they exhibited *TP53* mutations (including single-base substitutions, insertions, and deletions). Approximately 28% of BRCA tumors displayed *TP53* mutations on one allele in conjunction with a partial or complete 17p deletion on the other allele (Figure 5B). Interestingly, a notable portion of *TP53*-altered tumors exhibited chromosome 17p deletion despite having 1 WT *TP53* allele. Subsequently, we also assessed the *MYH4* status in these tumors. The analysis unveiled that in cases where *TP53* was deleted, there was a predominant co-deletion of *MYH4* (due to complete or segmental deletions; Figure 5B). Similar trends were observed in pan-cancer tumor samples from the PCAWG dataset (Supplemental Figure 5, A and B). Given the prior findings from Lowe’s lab (22, 52), we proceeded to test the hypothesis that *MYH4* loss could contribute to tumorigenesis together with *TP53* loss.

To investigate this, we utilized a somatic mouse model that permits in vivo assessment of the impact of loss of a gene of interest on p53-deficient mammary tumorigenesis (53, 54). This model is based on the intraductal injection of lentivirus encoding Cre recombinase and a single guide RNA (sgRNA) against the gene of interest (LentiCre-sgRNA) into the mammary gland of Cas9-expressing *Rosa26-Cas9*; *Trp53^fl/fl^* mice to induce concurrent Cre-mediated deletion of *Trp53* and Cas9-mediated disruption of the gene of interest in mammary gland epithelial cells. To test the effect of *Myh4* loss, *Rosa26-Cas9*; *Trp53^fl/fl^* females were intraductally injected with LentiCre-sgRNA against *Myh4*, *Atm* (as a positive control), or nontargeting (NT) sgRNA (inducing loss of *Trp53* alone) in groups of 8 mice per gene (Figure 5C). Both NT and *Myh4* groups experienced the loss of 1 mouse each before the start of the experiment.

First, we analyzed the overall survival of the groups (described in Methods). As demonstrated in Figure 5D, concomitant inactivation of *Myh4* and *Trp53* or *Atm* and *Trp53* led to faster mammary tumor development than inactivation of *Trp53* alone, shown as median overall survival times of 299 days for the *Myh4* group, 273 days for the *Atm* group, and 490 days for the NT control group. Both the *Atm* and *Myh4* groups exhibited significantly shorter overall survival compared with the NT group (*P =* 0.01794 and *P =* 0.00641, respectively). The overall survival curve for the *Atm* group indicated a 5-fold increased risk of tumor events, whereas the *Myh4* group indicated an 8-fold increased risk, relative to the NT group. We did not observe significant differences in tumor-free survival of *Atm* (*P =* 0.426) or *Myh4* (*P =* 0.216) when compared to NT mice, with median tumor-free survival of 241 days for the *Myh4* group, 244 days for the *Atm* group, and 319 days for the NT control group (Supplemental Figure 5C). This indicates that *Myh4* might not be contributing to tumor onset per se, but rather aggravates the cancer progression.

Next, to evaluate the nature of these observed tumors, we performed molecular and histopathological analysis. To do this, PCR analysis of the *Trp53^fl/fl^* alleles was conducted in 2 sg*Myh4*-injected and 1 NT tumor to confirm Cre-mediated *Trp53* deletion (Supplemental Figure 5D). To assess disruption of the targeted gene, tracking of indels by decomposition (TIDE) analysis was conducted in the largest tumor of each mouse, as indicated in Supplemental Figure 5E, with the total effect (%) of small insertions and deletions (indels) frequency. Next, for tumor type scoring we performed histopathological analysis of the largest tumor of each mouse, with hematoxylin and eosin (H&E) staining (Figure 5E and Supplemental Figure 5, F and G). We exclusively observed sarcomas in the main tumors of the NT group, while in the *Myh4* group we observed adenocarcinomas and sarcomas and in the *Atm* group, mixed carcinoma and sarcomas were observed. We focused this analysis on fully advanced tumors, excluding initial lesions. This analysis suggests that the loss of *Myh4* resulted in a subtle phenotypic change, leading to a spectrum of tumor types that may indicate a broader cellular origin of tumorigenesis compared with NT tumors.

Altogether, our data provide direct in vivo evidence that suppression of *Myh4* in conjunction with *Trp53* loss induces more aggressive disease.

## Discussion

In this study, we combined genetic and functional genomic screens with cellular and in vivo studies to identify breast cancer suppressor genes. We identified a genome maintenance function for MYH4, a class II myosin family protein. We functionally demonstrated its role in facilitating faithful replication, maintaining genomic integrity and limiting cancer progression.

### A cohort-informed approach toward genes with cryptic links to cancer.

As a starting point, we searched for candidate genes through germline sequencing of patients with early-onset breast cancer (23). We hypothesized that identified, mutated genes could play a role in genome maintenance pathways akin to BRCA1 (6).

### MYH4: a nonconventional DDR gene.

We selected *MYH4* as a focus of this study for several reasons, including its location on chromosome 17p near *TP53*. In addition, *MYH4* scored robustly in cell fitness screens in both cell lines, and links with genome maintenance have not been described. We found that MYH4 supported genomic integrity and its loss led to elevated levels of γH2AX in an ABD-dependent manner. Our functional analysis of MYH4 revealed that replication-associated challenges preceded DNA damage response signaling following its suppression. Altogether, these findings demonstrate a role for MYH4 in mitigating DNA replication stress, broadening its function beyond merely supporting cell fitness. Our findings extenuate earlier research indicating roles of myosins in DNA replication. Whether MYH4 operates in these capacities within the nucleus or in the cytoplasm will be an interesting question for future exploration.

### Loss of class II myosins in cancer.

Chromosome 17p harbors the *TP53* tumor suppressor gene and is frequently deleted in many cancers (51, 55–59). MYH4 and other class II myosins share structural similarities and, notably, several members of this class are located at the same genetic locus on chromosome 17p, implying a close genetic relationship among these myosin variants. Consistent with the DepMap analysis, our observations revealed a pattern of cell fitness codependency among various tested class II myosins with MYH4. We found that hypomorphic suppression of class II myosins was efficient in inducing replication-associated DNA damage and cell death. These codependencies may create vulnerabilities in cancer cells, potentially making them susceptible to targeted treatments that enhance replication stress (60). Furthermore, the identification of additional genes, particularly those within the actin-myosin pathway, that exhibit synthetic lethal interactions with MYH4 holds promise for the discovery and therapeutic exploitation of novel cancer vulnerabilities.

Consistent with prior genetic analyses, our survey indicated the loss of MYH4 due to segmental loss on chromosome 17p in breast tumors and various other cases. This may lead to cumulative haploinsufficiency and reduced gene dosage of class II myosins. A study from Lowe’s lab showed that somatic heterozygous deletion of the syntenic region on mouse chromosome 11 may accelerate MYC-driven lymphomagenesis through p53-independent mechanisms (22). Subsequent shRNA-based in vivo screening uncovered shRNAs targeting 17 genes in the chromosome 11 region, including the class II myosins *MYH1*, *MYH3*, and *MYH8*, which were enriched in the tumors, suggesting tumorigenic effects linked to the loss of these genes. Thus, the selective advantage resulting from the deletion of human chromosome 17p may be a consequence of both *TP53* loss and the reduced dosage of associated tumor suppressor genes. In line with this, our somatic *Myh4*-KO mouse mammary tumor model also displayed aggravated tumor formation induced by p53 loss. This suggests that *MYH4* loss contributes to acceleration of tumorigenesis beyond *TP53* loss alone. Furthermore, we observed that *Trp53* and *Myh4* inactivation altered the spectrum of cancers arising in the mouse model toward adenocarcinomas, rather than the sarcomas that are typical of p53 inactivation alone.

The Lowe lab’s screen also found an enrichment of shRNAs targeting Alox family genes (*Alox15b*, *Alox12b*, and *Alox3e*) in tumors. This observation suggests the intriguing possibility that the deletion of 17p may collectively diminish the activity of this entire gene family. In a similar way, we predict that the loss of heterozygosity of the chromosome 17p region may contribute to cumulative haploinsufficiency of myosin class II family genes, thus fueling tumorigenesis. While loss or dosage reduction of these genes individually may have a relatively modest impact on tumorigenesis, their combined effect might more strongly enhance tumor development upon *TP53* loss (61).

Segmental 17p loss can also include *POLR2A* that encodes the catalytic subunit of RNA polymerase II, and cancer cells with hemizygous *TP53* deletion are vulnerable to further suppression of *POLR2A* (59, 62). In a similar manner, tumors characterized by loss of *MYH4*/myosin class II genes and *TP53* might be vulnerable to further targeted suppression of class II myosins. Notably, the proposed role of MYH4 in replication stress suppression might also be therapeutically exploited. With this notion and our preliminary findings, it is tempting to speculate that tumors with loss of 17p will be vulnerable to drugs that enhance replication stress or prematurely advance cell cycle progression, such as WEE1, ATR, or CHK1 kinase inhibitors (47).

Collectively, our genetic and functional study of MYH4 provides valuable insights that highlight its role in cancer progression.

## Methods

### Sex as a biological variable

In our mouse models, sex was not considered as a biological variable because only female mice were used for cancer induction experiments. In the analysis of the PCAWG cohort, sex was not considered as a biological variable.

### Cell lines

Human osteosarcoma cell line (U2OS) and human embryonic kidney 293 cells (HEK293T) were grown in DMEM, supplemented with 10% FBS (HyClone, HYCLSV30160.03) and 1% penicillin/streptomycin (GIBCO, 15140-130). The human nonmalignant breast epithelial cell line MCF10A (WT and *TP53*-KO) (63) was cultured in DMEM/F-12 (GIBCO, 31330095) supplemented with 5% horse serum (GIBCO, 26050088), 1% penicillin/streptomycin, 10 μg/mL insulin (Sigma-Aldrich, I1882), 0.5 μg/mL hydrocortisone (Sigma-Aldrich, H0888), 20 ng/mL EGF (PeproTech, AF-100-15), and 100 ng/mL cholera toxin (Sigma-Aldrich, C8052). The human breast cancer cell line MCF7 was cultured in RPMI 1640 medium (Thermo Fisher Scientific, 61870036) supplemented with 10% FBS and 1% penicillin/streptomycin. All cell lines were from American Type Culture Collection (ATCC).

### Cohort and gene selection

Detailed information about the early-onset breast cancer cohort and whole-exome sequencing analysis has been published in a previous study (23).The present cohort was expanded with an additional 6 patients and consists of 135 patients. The filtering process was carried out as follows: variants with an allele frequency greater than 1% in public variant databases, including the 1000 Genomes Project (www.1000genomes.org) and the Genome Aggregation Database (gnomAD; http://gnomad.broadinstitute.org), were excluded, except for those variants already established as pathogenic despite being common. This approach aligns with previously described methods (23). Control cohorts were not included in this analysis, as association testing was not part of the filtering strategy.

We shortlisted all genes with at least one loss-of-function and one missense variant across the genome, resulting in 150 genes for downstream assessment using siRNA screening. Based on this analysis, *MYH4* was selected for further functional analysis and identified variants from the cohort are listed in Supplemental Table 1.

### Arrayed cell fitness screen

The siRNA library, which targeted 150 specific genes (list provided in the Supplemental Datasets 1 and 2), was acquired from Ambion (Silencer Select siRNA). Each gene was subjected to targeting by 3 distinct siRNAs, and their location in the 384-well plate was randomized using an Echo 550 liquid dispenser (Labcyte) to prevent positional bias. The arrayed siRNA screening was conducted in an automated manner utilizing the Hamilton STARlet liquid dispenser. On day 0, MCF10A and U2OS cells were reverse transfected using 10 nM silencer select siRNA. Five days after transfection, the cells were fixed in a 4% formaldehyde solution for 15 minutes. Subsequently, the fixed cells underwent 4 PBS washes and were permeabilized with 0.25% Triton X-100 for 10 minutes. Following permeabilization, the cells were washed 4 more times with PBS and were then exposed to 1 μg/mL DAPI for 30 minutes at room temperature. Following DAPI treatment, cells were subjected to 5 additional PBS washes and then imaged using an IN Cell Analyzer 2200 microscope with a 20× objective. All subsequent analyses were conducted using the IN Cell Analyzer Workstation (Cytiva) software. DAPI staining facilitated the identification of nuclei through the tophat segmentation method, which was used as a readout for cell fitness screening.

### siRNA transfection

siRNAs were synthesized by Sigma-Aldrich and reconstituted in Tris-EDTA buffer solution (Sigma-Aldrich, 93283) at 20 μM concentration. Transfection was performed using Lipofectamine RNAiMAX (Thermo Fisher Scientific, 13778500). All siRNAs were used at 10 nM, unless specified otherwise. Transfections were performed for either 48 or 72 hours as specified in figure legends. The siRNA sequences used in this study are provided in Supplemental Table 2.

### Molecular cloning

The pcDNA3.1(+)-eGFP plasmid containing the human *MYH4* cDNA, tagged with eGFP at the C-terminus, was synthesized by GenScript Biotech and subsequently re-cloned into the pLVX-TetOn-Puro lentivirus vector using BamH1 and EcoRI restriction enzyme sites by following the InFusion cloning technique (Takara Bio). Generation of siRNA-resistant MYH4 was achieved using PCR amplification (KOD hot start polymerase). Mutagenesis and plasmids were confirmed by Sanger sequencing. Primer sequences used for mutagenesis are listed in Supplemental Table 3. The ΔNeck and ΔC-ter mutants were synthesized by GenScript Biotech.

### Generation of stable MYH4-expressing cell lines

In order to create a stable U2OS cell line that expresses MYH4 in an inducible manner, HEK293T cells were transfected with 3 μg of either WT or mutant pLVX-MYH4-C-GFP plasmid, 1 μg VSV-G (Clontech), and 1 μg PAX2 (Clontech) plasmids using JetPEI to produce lentivirus according to the manufacturer’s protocol. The culture media were changed 5 hours after transfection. Twenty-four hours after transfection, the supernatant was collected by centrifugation at 300*g* for 5 minutes to obtain lentiviral particles. Five milliliters of the supernatant containing lentiviral particles was mixed with 5 mL of fresh U2OS medium containing polybrene (Sigma-Aldrich, H9268) at 10 μg/mL and added to the U2OS cells. Twenty-four hours after transduction, MYH4-GFP–containing U2OS cells were selected using 3 μg/mL puromycin for 10 days. Cells were induced with 1 μg/mL DOX for 16–24 hours, and sorted using a BD FACSMelody to ensure the presence of a moderately MYH4-GFP–expressing population. The expression was verified by immunoblotting and immunostaining (Supplemental Figure 2, A and B).

### Immunostaining

Cells were grown in 96-well mircoplates (Greiner-BIO) or on 12-mm coverslips. To study chromatin-bound proteins, cells were pre-extracted prior to fixation using pre-extraction buffer (25 mM HEPES, pH 7.5, 50 mM NaCl, 1 mM EDTA, 3 mM MgCl_2_, 300 mM sucrose, and 0.5% Triton X-100) on ice for 5 minutes and immediately fixed using 4% formaldehyde at room temperature for 15 minutes. For PCNA staining only, cell were fixed using prechilled methanol for 5 minutes and subsequently 10 minutes with paraformaldehyde at room temperature. After fixation, cells were permeabilized using 0.25% Triton X-100 in PBS at room temperature for 5 minutes. The permeabilization step was skipped for pre-extracted cells. The samples were blocked using blocking buffer containing 1% BSA, 0.1% Triton X-100, and 0.15% glycine in PBS for 1 hour at room temperature. Primary antibodies were diluted using the same blocking buffer and cells were stained at room temperature for 1 hour, followed by 3 washes with 0.05% Tween 20 in PBS. The samples were then incubated with fluorescently labeled secondary antibodies (Alexa Fluor dyes, 1:1000; Thermo Fisher Scientific) for 30 minutes and DAPI (1 mg/mL; Sigma-Aldrich) for 5 minutes at room temperature. Cells were stored in PBS at 4°C until imaging.

Primary antibodies used in this study were rabbit anti-γH2AX (1:1000; Cell Signaling Technology, 2577) or mouse anti-γH2AX (1:1000; Millipore, MABE285), anti–p-RPA (S33) (1:500; Novus, NB100-544), mouse anti-MCM2/BM28 (1:500; BD Biosciences, 610700), rabbit anti–p-MCM2 (S40/S41) (1:500; Bethyl Laboratories, A300-788A), and rabbit anti-PCNA (1:500; Abcam, ab18197).

### EdU incorporation–Click-iT assay

To detect the S phase, cell were pulsed with 10 μM EdU and chased for 30 minutes at 37°C. For EdU detection, after fixation and permeabilization, cells were subjected to Click-iT reactions according to the manufacturer’s protocol (Invitrogen Click-iT EdU) at room temperature for 45 minutes. Alexa Fluor 594 Azide was used a secondary reagent for detection (Thermo Fisher Scientific, A10270). The cells were washed 3 times with PBS and subsequently used for immunostaining.

### Microscopy and QIBC

Images used for QIBC were obtained with the ScanR (Olympus) acquisition software controlling a motorized Olympus IX-83 wide-field microscope. The QIBC data presented in this study were obtained using an Olympus Universal Plan Super Apo 20× objective. The acquired images were processed using the ScanR image analysis software. TIBCO Spotfire software (PerkinElmer) was utilized to create scatter diagrams, where total nuclear pixel intensities and mean nuclear intensities were plotted for DAPI and other parameters as specified in the corresponding figure legend. The *x* axis represents DNA content, indicated by total DAPI intensities on a logarithmic scale. The *y* axis displays mean intensities for respective antigens, also on a logarithmic scale.

Each scatter plot visualized more than 2000 random cells. The color gradients and thresholds applied in the QIBC scatter plots aimed to enhance the visual distinction of intensity differences among the experimental conditions.

Confocal images were taken on a Zeiss LSM 800 confocal microscope using a 63× objective (Zeiss Plan-Apochromat 63×/1.3 Oil DIC). Representative images were further analyzed using ImageJ software (NIH).

### DNA fiber assay

The U2OS cells were seeded in a 6-well plate at approximately 70% confluence. The following day, the cells were pulsed with CldU at a concentration of 25 μM for 30 minutes. After that, they were washed 3 times with PBS and then subjected to a second pulse using a different label, IdU, at a concentration of 250 μM for 1 hour. The labeled cells were harvested in ice-cold PBS. Four microliters of the cell suspension was placed on Superfrost slides (Thermo Fisher Scientific) and mixed with 8 μL of lysis buffer (0.5% SDS, 200 mM Tris pH 7.5, 50 mM EDTA) and incubated for 2 minutes. The mixture was then placed on superfrost slides, tilted at an angle of 15°–20° to allow the cell lysate to flow slowly along the slide, air-dried for 10 minutes, and fixed in methanol/acetic acid (3:1) for 10 minutes. After brief PBS washes, the slides were incubated in denaturation buffer (2.5 M HCl: 51 mL 1N HCl [which is 12 M] + 200 mL H_2_O) for 80 minutes.

After that, slides were blocked in blocking buffer (1× PBS, 0.1% Triton X-100, 1% BSA) for 30 minutes. For CldU detection, a rat anti-BrdU antibody (1:200; Abcam, ab6326) was applied to the slides in the blocking buffer for 75 minutes at room temperature. The slides were then washed once with PBS containing 0.1% Tween 20, followed by 2 additional washes with PBS. Next, the slides were fixed with 4% formaldehyde and incubated with an Alexa Fluor 568–conjugated anti-rat secondary antibody (1:100; Life Technologies) for 1 hour. Subsequently, the slides were washed with PBS, and IdU was detected using a mouse anti-BrdU antibody (1:200; BD Biosciences, 347580) overnight at 4°C, followed by incubation with an Alexa Fluor 488–conjugated anti-mouse secondary antibody (1:100; Life Technologies) for 1 hour. The images were acquired with a Zeiss Axio Imager M2 microscope at ×63 magnification, and the statistical analyses were performed using ImageJ software. A minimum of 200 fiber tracks were analyzed for each experimental condition.

### Cell viability assay

Following siRNA treatment for 24 hours, U2OS cells were reseeded in 96-well plates in triplicate and treated with either DMSO or WEE1 inhibitor (Debio 0123, Selleck, S9778) for 48 hours. Cell viability was assessed using a Cell-TiterGlo assay kit (Promega, G9241) according to the manufacturer’s protocol and luminescence was measured using a Molecular Devices SpectraMax iD3.

### Alkaline comet assay

Following siRNA treatment for 48 hours, U2OS cells were harvested at a concentration of 1 × 10^5^ cells/mL in ice-cold PBS. The alkaline comet assay was performed using the COMET assay kit according to the manufacturer’s protocol (Trevigen, 4250-050-K). The cells were embedded in low-melting-temperature agarose and transferred onto a Gel Bond Film (Lonza, 53734). The samples were placed at 4°C in order to allow the agarose to solidify. Subsequently, the samples were lysed using lysis buffer (R&D Systems/Bio-Techne Brand, 4250-050-01) overnight at 4°C. The following day, electrophoresis was carried out at 30 V on ice for 30 minutes. Afterward, the samples were treated with SYBR Gold Nucleic Acid Stain (Invitrogen, S11494), and images were captured using a Zeiss Axio Imager M2 microscope with a 10× objective. The comet tail moment was analyzed using the OpenComet plugin in ImageJ software.

### Real-time RT-PCR

The extraction of total RNA was performed using the RNeasy Mini Kit (QIAGEN, 74106) following the manufacturer’s instructions. Subsequently, cDNA synthesis was carried out using 500 ng of total RNA with the RT2 First Strand Kit (QIAGEN, 330404). For amplification, 10 ng of cDNA was used with the Maxima SYBR Green/ROX qPCR Master Mix (Thermo Fisher Scientific, K0221) on a LightCycler 480 instrument (Roche). The relative mRNA expression levels were determined using the ΔΔCt method, with *GAPDH* serving as the reference gene. The qPCR primer sequences employed in this study can be found in Supplemental Table 4.

### Cell fractionation

To obtain soluble and chromatin extracts, the following procedure was employed: cells were seeded in 10-cm dishes and treated as specified in the corresponding figure legends. After that, they were washed 3 times with ice-cold PBS and collected by trypsinization. The soluble fraction was extracted by incubating the cells in ice-cold nuclear buffer (10 mM HEPES pH 7, 200 mM NaCl, 1 mM EDTA, 0.5% NP-40) supplemented with protease inhibitors (cOmplete, EDTA-free Protease Inhibitor Cocktail, Roche, 11873580001) and phosphatase inhibitor (PhosSTOP, Roche, 4906845001) for 10 minutes on ice. Subsequently, the mixture was centrifuged at 2000*g* for 6 minutes. The resulting pellet was then rinsed once with ice-cold washing buffer (10 mM HEPES pH 7, 50 mM NaCl, 0.3 M sucrose, 0.5% Triton X-100) supplemented with protease and phosphatase inhibitors. The buffer was removed by centrifugation at 1400*g* for 6 minutes. Finally, the chromatin fractions were obtained by incubating the pellet in RIPA buffer (Sigma-Aldrich, R0278) supplemented with protease and phosphatase inhibitors as well as Benzonase Nuclease (Sigma-Aldrich, 70746-4) for 30 minutes on ice. The mixture was then clarified by centrifugation at maximum speed.

### Immunoprecipitation and mass spectrometry

In a 15-cm dish, the stable expression of MYH4-GFP in the U2OS cell line was induced by adding 1 μg/μL DOX for 24 hours. The next day, cells were trypsinized and washed with PBS. Cell pellets were incubated in RIPA buffer supplemented with protease and phosphatase inhibitors as well as Benzonase Nuclease for 30 minutes on ice. The mixture was then clarified by centrifugation at maximum speed. Protein concentration was measured with Bradford Assay. Protein lysate (500 μg) was used further for immunoprecipitation. GFP-Trap beads (ChromoTek, gta-100) were equilibrated by suspending 25 μL of bead slurry per immunoprecipitation reaction in 500 μL of RIPA lysis buffer. The beads were washed twice with lysis buffer. The protein lysate was added to the equilibrated GFP beads and incubated at 4°C for 2 hours with constant mixing. Meanwhile, input samples were prepared by adding the required amounts of protein lysate, 4× Laemmli sample buffer (Sigma-Aldrich), and lysis buffer. Following incubation, the tubes were centrifuged at 2500*g* for 2 minutes at 4°C, and the supernatant was discarded. The beads were washed at least 3 times with 1 mL of lysis buffer, ensuring minimal disturbance to the settled beads during the first 2 washes.

For Western blot analysis, the GFP-Trap beads were resuspended in 30 μL of 4× Laemmli sample buffer and boiled for 10 minutes at 95°C. The immunocomplexes were dissociated from the beads, and the supernatant was collected for SDS-PAGE.

For mass spectrometry analysis, the beads were washed 3 times with PBS and resuspended in guanidinium chloride. The samples were then prepared for on-bead digestion following a specific mass spectrometry protocol (64).

### Western blotting

To obtain whole-cell extracts, cells were lysed in RIPA buffer containing EDTA-free protease inhibitor cocktail and phosphatase inhibitors. The lysates were then treated with Benzonase Nuclease on ice for 30 minutes. After centrifugation at 20,000*g* for 15 minutes at 4°C, the protein concentration of the lysates was assessed using the Bradford assay. The lysates were mixed with 4× Laemmli sample buffer and boiled at 95°C for 10 minutes. Subsequently, the samples were loaded onto NuPAGE Bis-Tris 4%–12% gels (Thermo Fisher Scientific, NP0323BOX) following the manufacturer’s instructions. Proteins were transferred to a nitrocellulose membrane and blocked with PBS with 0.1% Tween 20 and 5% skim milk powder (Sigma-Aldrich). The membrane was then incubated overnight at 4°C with the primary antibodies diluted in the same blocking buffer. Subsequently, the membrane was washed 3 times for 5 minutes each with PBS plus 0.1% Tween 20 and incubated with HRP-conjugated secondary antibodies for 1 hour at room temperature. Following another round of washing, the membrane was incubated with Classico/Crescendo Western HRP substrate (MilliporeSigma) for 1 minute, and the chemiluminescence signal was detected using a Bio-Rad ChemiDoc Touch Imaging System. Some of the blots were reblotted after stripping the first antibody with ReBlot Plus Strong solution (Merck-Millipore, 2504).

The following primary antibodies were used: rabbit anti-MYH4 (Novus, NBP3-05634), rabbit anti-GFP (Abcam, ab290), rabbit anti-OCR1 (Abcam, ab85830), mouse anti-MCM2/BM28 (BD Biosciences, 610700), rabbit anti–p-MCM2 (S40/S41) (Bethyl Laboratories, A300-788A), rabbit anti-CDK2 (Cell Signaling Technology, 2546), rabbit anti-PCNA (Abcam, ab18197), mouse anti-vinculin (Sigma-Aldrich, V9131), mouse anti-actin (Merck-Millipore, MAB1501), rabbit anti–histone H4 (Millipore, 05-858), rabbit anti-CDC6 (Cell Signaling Technology, 3387S), and rabbit anti-CDT1 (Cell Signaling Technology, 8064S).

### Synergy analysis

A dose-response matrix experiment was conducted using 5 different concentrations of siRNA (0, 2, 6, and 10 nM) targeting MYH4, along with the knockdown of 8 other genes across the same concentration range. The siUNC served as the normalization control, aiming for a final concentration of 20 nM per well. The assay plates were structured to incorporate a randomized arrangement of all samples, using Python programming (https://www.python.org/). The Echo 550 acoustic liquid handler facilitated the precise dispensing of small volumes of siRNA; 3 wells were used per condition. A mixture of 10 μL RNAiMAX and opti-MEM was distributed to the wells using the MultiFlo FX dispenser (BioTek), followed by a 15-minute incubation at room temperature (with a final RNAiMAX concentration of 1:1000). The plates were centrifuged at 180*g*. Subsequently, 40 μL of cell suspension containing 600 cells per well was dispensed using the MultiFlo FX dispenser. The plates were centrifuged for 1 minute and were then incubated at 37°C for 5 days. A solution of 10 μL CellTox Green (1:5000; Promega, G8731) and Hoechst stain (1:1000; Thermo Fisher Scientific, H3570) in PBS was added to the plates using the MultiFlo FX dispenser and incubated at 37°C. Whole-well images were captured using an ImageXpress Confocal HT.ai with 4× and 10× objectives. The percentage of cell death per condition was calculated by gating out the CellTox Green–positive cells (representing dead cells). Three siUNC wells were used to normalize the data and SynergyFinder 2.0 (https://synergyfinder.org/) was used to visualize the data.

To evaluate the synergy between different class II myosins, we utilized the Bliss independence model. This model is based on the assumption of a stochastic process in which the effects of 2 genes are independent of each other. In this model, the expected effect of the gene combination can be calculated by considering the probabilities of the independent events. A bliss score of 10 or higher indicates synergy, while a score of 10 or lower indicates antagonism. Using the SynergyFinder 2.0 tool, we calculated Bliss scores for each combination of genes at 6 nM.

### Analysis of the PCAWG cohort

Somatic small-scale variants and copy number alterations generated by the ICGC/TCGA PCAWG consortium (50) were downloaded from cBioPortal (https://www.cbioportal.org/study/summary?id=pancan_pcawg_2020 Accessed December 20, 2023). Somatic small-scale mutations, such as singe nucleotide variants, insertions, and deletions, were assessed by InterVar (65) to interpret the clinical significance of these sequence variants. InterVar classifies variants into 5 categories: “Benign,” “Likely Benign,” “Uncertain Significance,” “Likely Pathogenic,” and “Pathogenic.” Small-scale mutations classified as Pathogenic or Likely Pathogenic were considered when we determined *TP53* somatic mutation status (TP53 MUT). Variants of uncertain significance were noted as well, but they did not affect the genotyping scheme, i.e., they were marked as TP53 WT. Consensus putative gene-level copy-number calls determined by GISTIC 2 (https://broadinstitute.github.io/gistic2/), as described previously (50), were used to identify large-scale deletions affecting *TP53*, *MYH4*, and other myosins on chromosome 17p. The analysis was performed using the statistical and visualization software R (https://www.r-project.org/).

### Mouse studies

#### In vitro.

Tests performed on LentiCre-sgRNA–transduced mouse embryonic stem cells (Rosa26CreERT2/Cas9;*Trp53^–/–^*) showed successful CRISPR activity at the targeted sites. Cre activity was also observed when viruses were tested in HEK293 Cre-GFP reporter cells.

#### sgRNA design.

The sgRNAs were selected from 2 libraries: Mouse Improved Genome-wide Knockout CRISPR Library v2 (Yusa lab) (Addgene, 67988), sg*Myh4* (GAGTTCAGACTTGTCAGATA); and Mouse CRISPR Knockout Pooled Library (Brie) (Addgene, 73632), sg*Atm* (GAGTATAAATAACATCGCGA).

#### Lentiviral vectors.

The NT sgRNA (TGATTGGGGGTCGTTCGCCA) and the sgRNAs targeting *Myh4* and *Atm* were cloned as described previously (66) into the PLentiCre vector. To generate this vector, Cre-T2A was inserted into the lentiGuide-Puro vector (Addgene plasmid 52963) between the EF-1α promoter and the puromycin resistance fragment. The vector was validated by Sanger sequencing. We produced concentrated stocks of VSV-G pseudotyped lentivirus by transient cotransfection of 4 plasmids in HEK293T as described previously (67). The lentiviral titers were determined using the quantitative PCR (qPCR) lentivirus titration kit from Abm (LV900).

#### Somatic mouse models.

To generate *Myh4*- or *Atm*-deficient tumors in combination with the loss of *Trp53*, 8-week-old *Rosa26-Cas9*; *Trp53^fl/fl^* female FVB/n inbred mice (68, 69), genotyped as previously described (70, 71), were intraductally injected with lentiviruses encoding sg*Myh4*, sg*Atm*, or sgNT, in combination with Cre as described previously (72, 73). Briefly, 20 μL of high-titer lentiviruses (approximately 2 × 10^8^ transfection units per mL) were injected into the third and fourth mammary glands by using a 34-gauge needle. Mice were monitored 3 times per week for tumor development by palpation. Mammary tumor size (length and width in millimeters) was measured using calipers and tumor volume (mm^3^) was calculated using the following formula: 0.5 × length × width^2^. Mice were sacrificed when tumors reached a size of 1500 mm^3^ or when reaching the humane endpoint. Mice were bred and maintained in accordance with institutional, national, and European guidelines for animal care and use.

#### Histology.

Tumors were formalin-fixed overnight and paraffin-embedded (FFPE) by routine procedures. H&E staining was performed as described previously (74). H&E slides were used to classify mammary tumor lesion types. They were reviewed by a comparative pathologist in a blinded manner.

#### PCR amplification and TIDE analysis.

To perform this analysis, a random piece of the tumor was used as starting material. The amplification of *Myh4* exon 13 and *Atm* exon 29 was performed with specific primers spanning the target sites (FW *Myh4*: TTGCCATCACTGGGATAGGG; RV *Myh4*: ACGTGACTGCTAAAGTGCATC; FW *Atm*: GAGGTTACCGAAGACCCACG; and RV *Atm*: CCAGGGCTGTTACACAGCGAG) and 1 μg of DNA template using the Q5 high-fidelity PCR kit from New England Biolabs. Amplicons were purified using the Isolate II PCR and Gel kit (Bioline). PCR products were Sanger sequenced using the FW primer for *Myh4* and the RV primer for *Atm*, and CRISPR/Cas9-induced editing efficacy was predicted and quantified with the TIDE algorithm (http://tide.nki.nl/), as described previously (75).

#### Statistical analysis of mouse study.

The analysis of the survival data was done separately for tumor-free survival and overall survival as response, using the same approach in each case: Cox’s proportional hazards model was fitted to the response using the group (*Atm* and *Myh4* each compared to the reference group NT) as covariate. We also display the survival curves of the 3 different groups.

#### Inclusion and censoring of animals in statistical studies.

For overall survival analysis, in the NT group, 3 animals out of 7 contributed to the results. Of the remaining 4 animals, 2 mice were found dead in their cage, of which none experienced the event of death due to a large tumor (≥1500 mm^3^), 1 was euthanized due to non–tumor-related health issues, and 1 mouse was still alive with no tumors at the study’s conclusion. Therefore, these mice were censored. In the *Atm* and *Myh4* groups, all mice developed large tumors, and thus contributed to the results. For the tumor-free analysis, tumors that were larger than 62.5 mm^3^ when palpated for the first time were deemed too large at the initial finding and therefore excluded from the analysis. In the NT group, 3 out of 7 mice developed tumors and were included in the tumor-free survival analysis and in *Atm* and *Myh4* groups, all mice developed large tumors, and were thus included in the analysis.

### Statistics

All the statistical tests were performed using GraphPad Prism 10. The details are provided in the figure legends for specific tests. All the figures were assembled in Adobe Illustrator. Supplemental Figure 1A, Figure 5C, and the graphical abstract were generated using BioRender (https://www.biorender.com/).

### Ethics approval and consent to participate

This study includes use of clinical data for secondary use and the record is kept on file. The study was approved by The Capital Region of Denmark (H-4-2010-050), the Danish Data Protection Agency (RH-2016-353, I-Suite no. 05097), and the Danish Breast Cancer Cooperative Group (jr. no. DBCG-2013-15).

This study also includes use of animal models and all experiments were approved by the Ethics Committee of The Netherlands Cancer Institute.

### Data availability

The source data and results for siRNA-based cell fitness screens related to Figure 1, A and B are attached as a Supporting Data Values file. Additional raw data will be provided on request.

## Author contributions

JT, CSS, AMDS, J Börcsök, TG, J Benada, XL, MB, MR, FCN, CB, and JJ conceptualized the study. JT, AMDS, J Börcsök, TG, J Benada, XL, MB, HVDG, JYS, RM, EMD, VP, MR, FCN, and JJ developed the methodology. JT, AMDS, J Börcsök, TG, J Benada, XL, MB, JYS, and EMD performed experiments. JYS analyzed the tumor samples. JT, AMDS, J Börcsök, TG, J Benada, XL, MB, VP, and EMD generated figures. CSS, CB, and JJ supervised the study. LT supervised and analyzed the fiber assay experiments. JT, AMS, and CSS wrote the original draft of the manuscript, which was edited by CSS, JJ, AMDS, J Börcsök, TG, J Benada, XL, MB, MR, and FCN**)**.

## Supplementary Material

Supplemental data

Supplemental data set 1

Supplemental data set 2

Unedited blot and gel images

Supporting data values

## Version 1 06/02/2025

Electronic publication
