# Supplementary material for: The class II myosin MYH4 safeguards genome integrity and suppresses tumor progression
Source: J Clin Invest. 2025 Jun 2;135(11):e188165. doi: 10.1172/JCI188165 (PMC12126247; doi:10.1172/JCI188165)
Supplement: Supplemental data [file jci-135-188165-s313.pdf]

Supplementary File for

A class II myosin MYH4 safeguards genome integrity and suppresses tumor progression

Thatte Jayashree *et al.*

*Corresponding author. Email: claus.storgaard@bric.ku.dk

This PDF file includes:

Supplemental Figures, 1-5

Supplemental Tables, 1-4

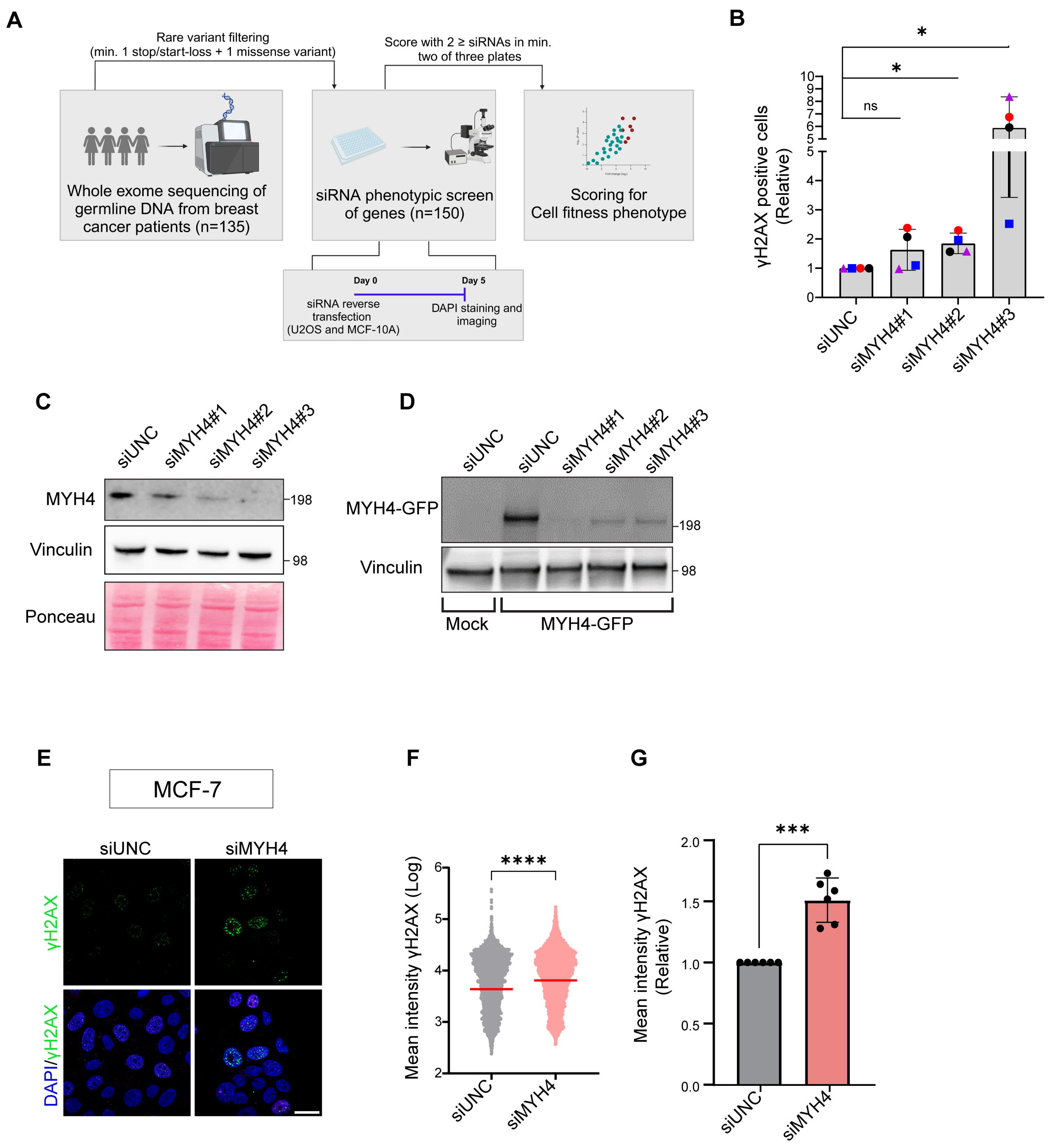

Supplemental Figure 1. Arrayed screen identifies MYH4 as a cell fitness and genome maintenance gene. (A) An outline of study including WES, gene filtering criteria, siRNA screening and gene scoring criteria. Bottom panel illustrates experimental design and timeline. (B) A bar chart showing relative γH2AX positive U2OS cells. Data points show 4 biological replicates with 3 different siRNAs against MYH4. Errors bars indicate standard deviation of mean (± s.d.). Statistical test: Two tailed paired t test, * = p-value < 0.05, ns = not significant. (C) A western blot analysis of whole cell extracts from U2OS cell line indicating depletion of endogenous MYH4 using 3 different siRNAs targeting MYH4, after 48h of transfection. The data points are from a single experiment, a representative of 3 independent biological experiments. (D) U2OS cells transfected with MYH4-GFP WT construct and either siUNC or 3 different siRNAs targeting MYH4 for 48h. Whole cell extracts were collected and analyzed by western blotting using antibody against GFP. It is a representative blot out of 3 independent biological replicates. *Note:* The siRNA#3 was chosen for further validations. (E) Representative confocal images of breast cancer cell line MCF7 showing γH2AX levels upon MYH4

knockdown. Scale bar is 20 µm. (F) Density plot of over 1000 cells (related to S1E), red line indicates median. Statistical test: Mann Whitney test, ****= p-value < 0.0001. (G) A bar chart showing relative γH2AX mean intensity of MCF7 cells transfected with siMYH4(#3) Data points show 6 biological replicates. Errors bars indicate standard deviation of mean (± s.d.). Statistical test: Two-tailed paired t test, *** = p-value < 0.001.

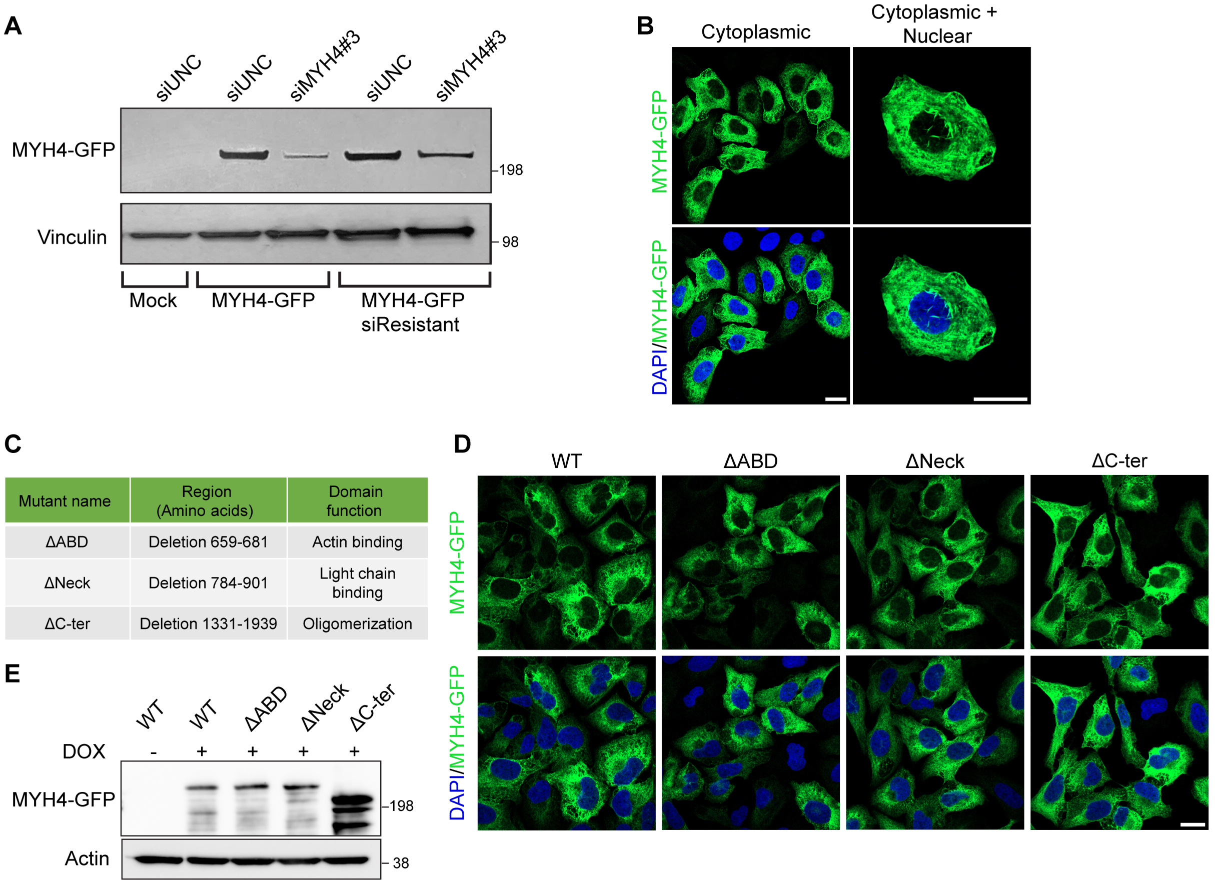

Supplemental Figure 2. MYH4, predominantly cytoplasmic protein, plays a role in maintaining genome integrity.

(A) A western blot analysis of whole cell extracts from cells transfected with either MYH4-GFP WT or siRNA resistant-MYH4-GFP WT construct together with either siUNC or siMYH4 for 48h. (B) Images from confocal microscopy depicting the localization of MYH4-GFP WT in the stable cell line, DAPI is used to stain nucleus. Scale bar is 20 µm. (C) A table indicating the amino acid region and domain function of each specified MYH4-GFP mutants. (D) Representative images showing localization of WT and indicated mutants of MYH4-GFP. Scale bar is 20 µm. (E) A western blot analysis of whole cell extracts from cells transfected with either MYH4-GFP WT or different mutants. Size: WT- 1939aa, ΔABD-1917aa, ΔNeck-1822aa, ΔC-ter-1331aa. (aa: Amino acids). N=3 for all above experiments.

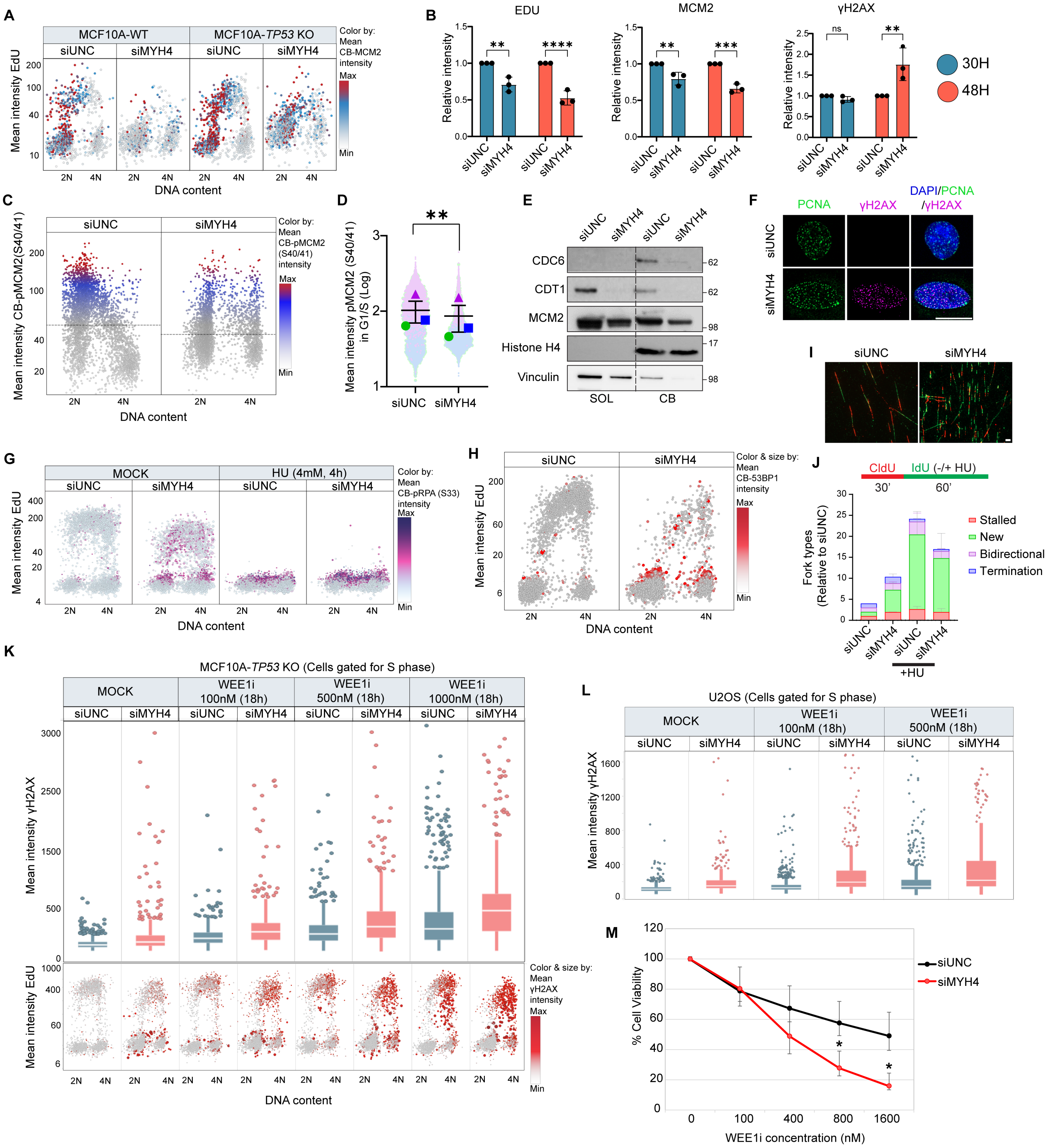

Supplemental Figure 3. MYH4 plays a role in ensuring replication licensing and facilitating replication. (A) QIBC of MCF10A-WT or *TP53* KO cells transfected with indicated siRNAs for 48h and stained for CB-MCM2. MCM2 distribution in different cell-cycle phases is shown. CB-MCM2 color threshold: Gray: Minimum, Blue: Average and Red: Maximum. (B) The bar charts showing relative intensities of EdU in S-phase in U2OS, CB-MCM2 in G1-early S phase and γH2AX respectively, of cells transfected with indicated siRNAs. Color indicates two time points: Blue: 30h, Red: 48h. The bar chart represents the means (± s.d.) of three independent biological replicates. Statistical test: Two-way ANOVA, Sidak's multiple comparison test; ** = p-value < 0.01, *** = p-value < 0.001, **** = p-value < 0.0001, ns= not significant. (C) QIBC of U2OS cells transfected with the indicated siRNAs and stained for CB phospho-MCM2 (S40/41). Mean intensity of phospho-MCM2 (S40/41) is shown, dashed line indicates average. The pMCM2 (S40/41) color threshold: Gray: Minimum, Blue: Average and Red: Maximum. (D) A density plot showing mean intensity of phospho-MCM2 (S40/41) from 3 biological replicates.. Errors bars indicate standard error of mean (± SEM). The p-values are from two-tailed paired t test, ** = < 0.001. (E) A western blot analysis of indicated pre-replicative complex

(pre-RC) and pre-initiation complex (pre-IC) proteins in SOL and CB extracts from U2OS cells transfected with indicated siRNAs. (F) Representative confocal images (related to Figure 3G) showing PCNA and γH2AX staining after 48h of indicated siRNA transfection. Scale bar is 20 µm. (G) QIBC of U2OS cells transfected with the indicated siRNAs and stained for CB-phosphoRPA (S33). The distribution of CB-pRPA (S33) in different cell-cycle phases is shown; CB-pRPA(S33) color threshold: Gray: Minimum, magenta: Average and purple: Maximum. (H) QIBC of cells transfected with the indicated siRNAs and stained for CB-53BP1. The distribution of CB-53BP1 in different cell-cycle phases is shown (indicated in red); CB-pRPA (S33) color threshold: Gray: Minimum, magenta: Average and purple: Maximum. (I) Representative Images of DNA fibers from U2OS cell line transfected with indicated siRNAs. Scale bar is 50 µm. (J) A bar plot of fork types identified with DNA fiber assay in U2OS cells transfected with indicated siRNAs, with or without HU treatment (0.5mM). The samples are normalized to siUNC to obtain relative values. Error bars indicate means (± SEM of 3 independent biological replicates. (K) Top panel: A box plot showing mean intensity of γH2AX in S phase of MCF10A-*TP53* KO cells transfected with indicated siRNAs for 48h and either untreated or treated with indicated WEE1i concentrations for last 18 h. Middle lines indicate medians. Outliers with intensity above 3000 are not displayed due to plot size constraint. The data points are from a single experiment, a representative of 3 independent experiments. Bottom panel: QIBC of MCF10A-*TP53* KO cells (related to top panel) stained for γH2AX. γH2AX distribution in different cell-cycle phases is shown. The γH2AX color threshold: Gray: Minimum, Blue: Average and Red: Maximum. (L) A box plot showing mean intensity of γH2AX in S phase of U2OS cells transfected with indicated siRNAs for 48h and either untreated or treated with indicated WEE1i concentrations for last 18 h. Middle lines indicate medians. The data points are from a single experiment, a representative of 3 independent biological experiments. (M) A Survival curve illustrating cell viability in U2OS cells transfected with indicated siRNAs for 72h and treated with WEE1 inhibitor for last 48h with indicated dose range. Error bars indicate means (± s.d.) of 3 independent biological replicates. Statistical test: t-test, *= p-value < 0.05. N=3 for all above experiments.

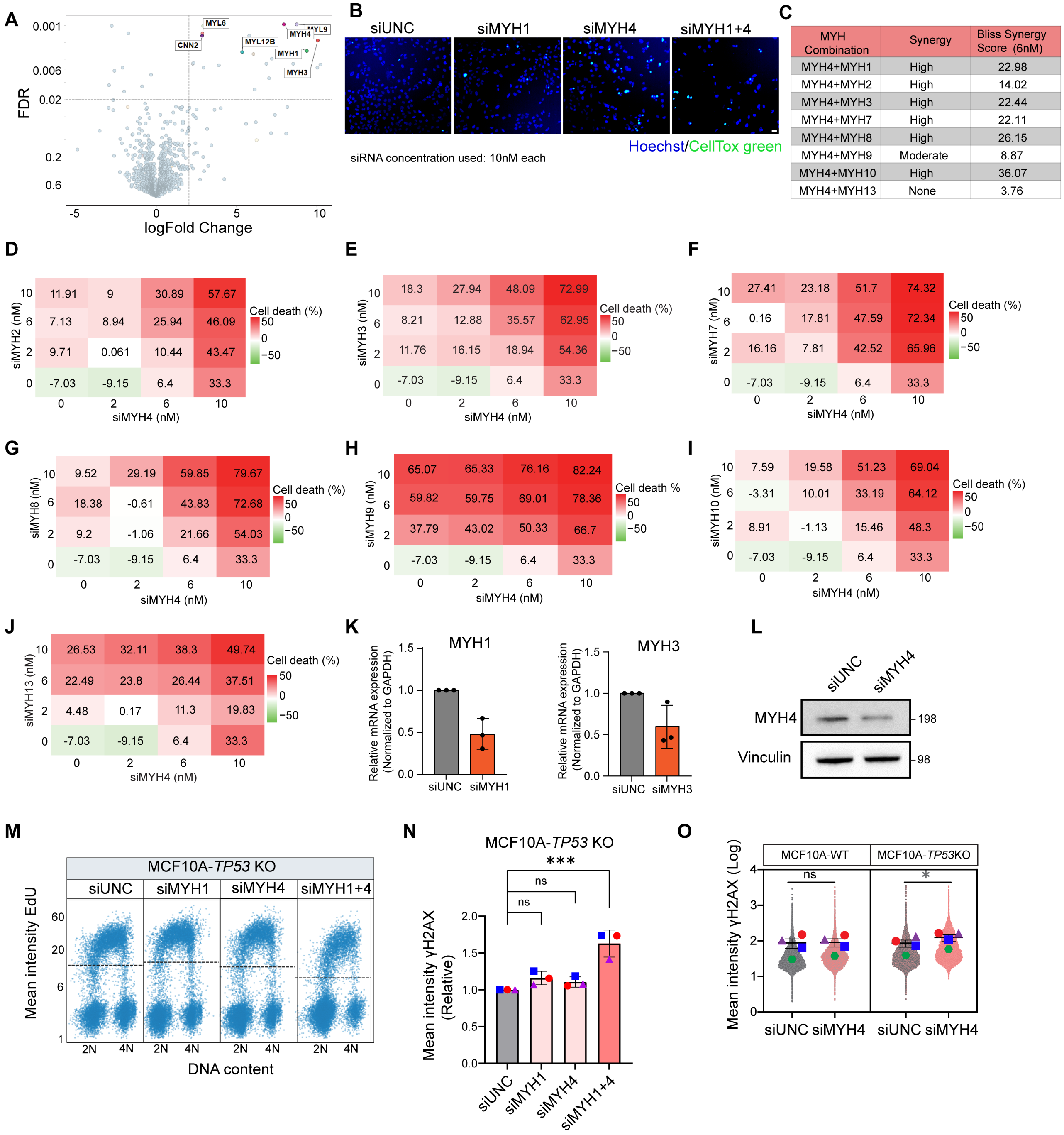

Supplemental Figure 4. Class II Myosins demonstrate co-dependency.

(A) Mass Spectrometry (MS) analysis of MYH4-GFP pulldown. Volcano plot of fold changes in protein levels is shown, highlighting proteins from myosin class II family. (B) Representative images of cells transfected with indicated siRNAs, (10 nM concentration) for 72h. Hoechst is used to stain nucleus and CellTox Green is used to stain dead cells. (C) A table indicating the Bliss synergy scores calculated for indicated class II myosins pairs, using 6 nM siRNA concentration. A score ≥10 represents synergy and a score ≤10 represents no synergy. (D to J) A matrix depicting the dose-response relationship for cell viability in cells following transfection with different concentrations indicated siRNA pairs for a duration of 72h. The values represent the mean of three replicates, normalized to three control values (siUNC) and calculated as a percentage of cell death. (K) The partial knockdown of MYH1 and MYH3 (related to Figure 4F) using 6 nM siRNA. The knockdown was assessed using qRT-PCR. The bar chart represents the means (± s.d.) of three independent biological replicates. Statistical test: Wilcoxon test for two paired samples, non parametric test, p-value: ns.(L) A western blot analysis of partial knockdown of MYH4 using 6 nM siRNA (Related to Figure 3F).

(M) QIBC plot indicating EdU incorporation of MCF10A- *TP53* KO cells transfected with the indicated siRNAs for 48h. Note that the siRNA concentration used for each siRNA is 6 nM for 48h. siUNC was used for transfection normalization to achieve a final concentration of 12 nM. Dashed line indicates average EdU intensity. (N) A bar chart showing relative mean intensity of γH2AX in MCF10A- *TP53* KO cells transfected with indicated siRNAs (6 nM each) for 48h. The data points are from 3 independent experiments, Statistical test: One way-ANOVA, multiple comparisons test, p-value ***= < 0.001, ns= not significant. (O) A density plot showing mean intensity of γH2AX in MCF10A- WT and *TP53* KO cells transfected with indicated siRNAs for 48h.The data points are from 4 independent experiments. The error bars indicate standard error of means (± SEM). Statistical test: Two-tailed paired t test, p-value * < 0.05, ns= not significant.

A

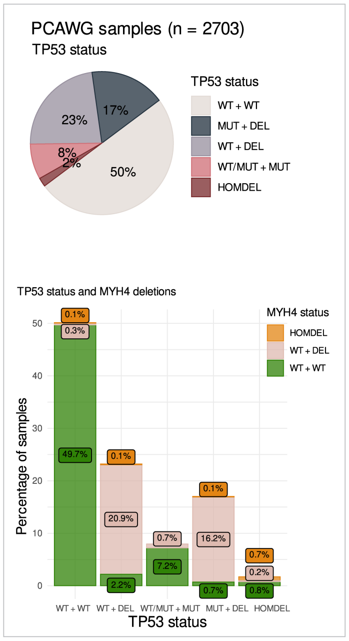

B

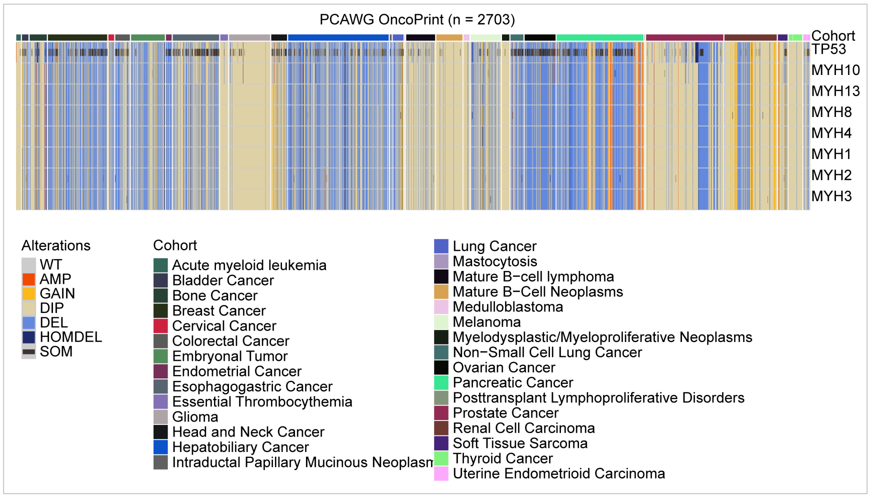

C

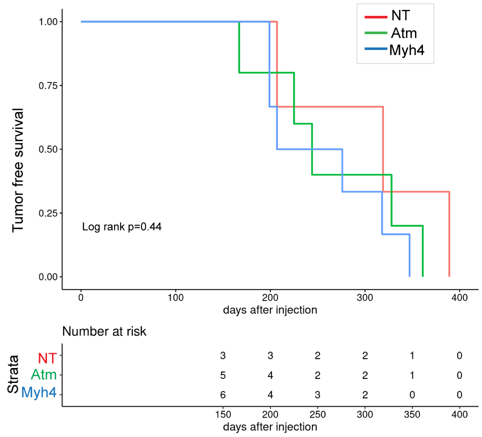

D

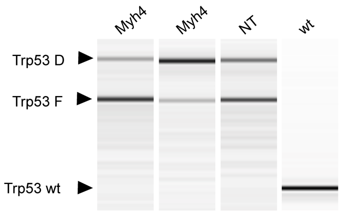

E

gene	#mouse	total editing effect (%)
Myh4	2295059	8.3
	2295061	8.9
	2295060	14.1
	2295058	19.2
	2295057	9.3
	2295056	10.8
	2295055	7.2
NT	2227220	4.6
	2312754	3.9
	2227222	4.8
Atm	2238032	2
	2249238	7.4
	2238033	7
	2249237	7.2
	2234643	7.1
	2249241	0.6
	2234644	7.4
	2227220	2.2
	2312754	1.7
	2227222	0

F

gene	#mouse	classification
NT	2227220	sarcoma
	2312754	sarcoma
	2227222	sarcoma
Myh4	2295059	adenocarcinoma
	2295061	sarcoma
	2295060	sarcoma
	2295058	sarcoma
	2295057	sarcoma
	2295056	adenocarcinoma
	2295055	sarcoma
Atm	2238032	sarcoma
	2249238	adeno carcinoma - squamous cell carcinoma
	2238033	sarcoma
	2249237	sarcoma
	2247354	sarcoma
	2234643	sarcoma
	2249241	sarcoma
	2234644	sarcoma

G

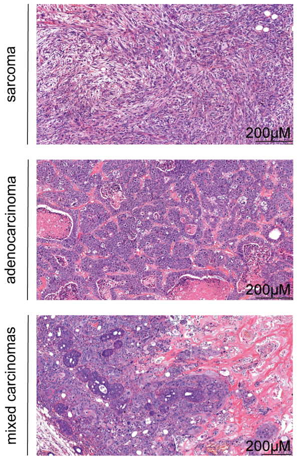

Supplemental Figure 5. *MYH4* loss accelerates tumorigenesis in *TP53* deficient background.
(A) Top panel: A pie chart depicting *TP53* mutation status of samples in the PCAWG cohort (n=2703), color code by *TP53* status. Bottom panel: Combined assessment of *TP53* and *MYH4* status in the PCAWG cohort, color code by *MYH4* status. (B) Oncoprint of samples in the PCAWG cohort (n = 2703) grouped by cancer type that shows small-scale pathogenic somatic variants and copy number changes in *TP53*, *MYH4* and other myosins on chromosome 17p. (C) Kaplan-Meier curves showing mammary tumor free survival of Rosa26-Cas9;Trp53$^{F/F}$ females injected with sgNT (n=3), sg*Atm* (n=5), and sg*Myh4* (n=6). Mice injected with sg*Atm*

and sg*Myh4* do not show a significant difference in tumorigenesis when compared to sgNT (p=0.44; log rank test). Number of animals at risk over time is represented in the table at the bottom.
(D) Representative *Trp53* PCR in two sg*Myh4*-injected and one NT tumors, showing *Trp53* deletion (D; 444 bp) and the Flox allele (F; 353 bp) band. The wild-type (wt) band (200bp) is observed at the control DNA (*Trp53*-proficient). (E) Total effect values (%) obtained from TIDE assays of the main tumor of each mice injected with sgNT (n=3), sg*Myh4* (n=7) or sg*Atm* (n=7). NT tumors were compared to *Myh4* and *Atm* guides. (F) Tumor type classification of the main tumor of each mice injected with NT (n=3), sg*Myh4* (n=7) or sg*Atm* (n=8). *Atm* group includes a subgroup, mixed carcinoma, where different carcinomas were found in the same lesion. In one specific case, a mix of adenocarcinoma and squamous cell carcinoma was observed. (G) Representative H&E staining in tumors of the three different tumor types present in the cohort: sarcoma (sg*Myh4*-injected; #2295061), adenocarcinoma (sg*Myh4*-injected; #2295059), and mixed carcinomas (sg*Atm*-injected; #2249238). Scale bar is 200 µm.

Supplemental Table 1. MYH4 variants found in Danish breast cancer cohort (n=135)

Nucleotide change	Protein Change	GnomAD frequency
MYH4.c.5491delG	p.Glu1831fs	0
MYH4.c.3798G>C	p.Lys1266Asn	0
MYH4.c.3339A>C	p.Lys1113Asn	0
MYH4.c.551G>T	p.Gly184Val	0.024

Supplemental Table 2. The siRNA sequences used in this study.

Gene Symbol	Sense siRNA Sequence
siUNC	UAACGACGCGACGACGUAATT
MYH4#1	GCAUAGCUCUCGAUAAGAATT
MYH4#2	CGAGUUACUUUUCAGCUAATT
MYH4#3	GAAGAACUCGAUCAGCUAATT
MYH1#1	GGUGAAGUUGAAAGUGAACTT
MYH1#2	GGAGUCCUUUGUGAAAGCATT
MYH1#3	GAAGGACAAUUCAUCGAUATT
MYH2	GGAAAAGUGACGGUGAAGATT
MYH3#1	GCAUAGAUGAUCGAGAGGATT
MYH3#2	GCUGAAAGCGAAAAUUUGUTT
MYH3#3	CCAGAGGAUGUGUACGCCATT
MYH7	CAAAUCCUGUCUAACAAAATT
MYH8	GCAACUCUAACUGUCAGGGTT
MYH9	AAGGAGCGUUACUACUCAGGG
MYH10	UAUUCUCAGAGUAAAUUGG
MYH13	GGAGAGAAUCGAGGCUCAATT

Supplemental Table 3. Molecular cloning primers.

Gene/mutant	Forward primer (5' to 3')	Reverse primer (5' to 3')
MYH4-GFP siRNA#3 resistant	GCTGAAAAAGATGAAGAgCTCGAcCAGCTgAAGAGGAACC	GGTTCCTCTTcAGCTGgTCGAGcTCTTCATCTTTTTCAGC
MYH4-GFP infusion cloning	CCCTCGTAAAGAATTCATGAGTTCTGACTCTGAGATGGCC	GAGGTGGTCTGGATCCTTACTTGTACAGCTCGTCCATG
MYH4-GFP ΔABD	[PHO] ACT CCT GGT GCC ATG GAG CAT GAG	[PHO] ATT CTC CCT GAA AAG AGC TGA CAC TGT C

Supplemental Table 4. The q(RT-PCR) primers.

Gene	Forward primer (5' to 3')	Reverse primer (5' to 3')
MYH1	CACCCTGACCAAAGCTAAAATC	TCTTCAATCTTGCTTTGCAGAC
MYH3	CTGGAGGATCAGTTAAGTGAGG	TTTCTTTTTCTTCCAGCTGACG
GAPDH	GGAGCGAGATCCCTCCAAAAT	GGCTGTTGTCATACTTCTCATGG
