# Supplementary material for: The class II myosin MYH4 safeguards genome integrity and suppresses tumor progression
Source: J Clin Invest. 2025 Jun 2;135(11):e188165. doi: 10.1172/JCI188165 (PMC12126247; doi:10.1172/JCI188165)
Supplement: Unedited blot and gel images [file jci-135-188165-s316.pdf]

Original uncropped western blot gel images:

Images were taken using BioRad Geldoc system.

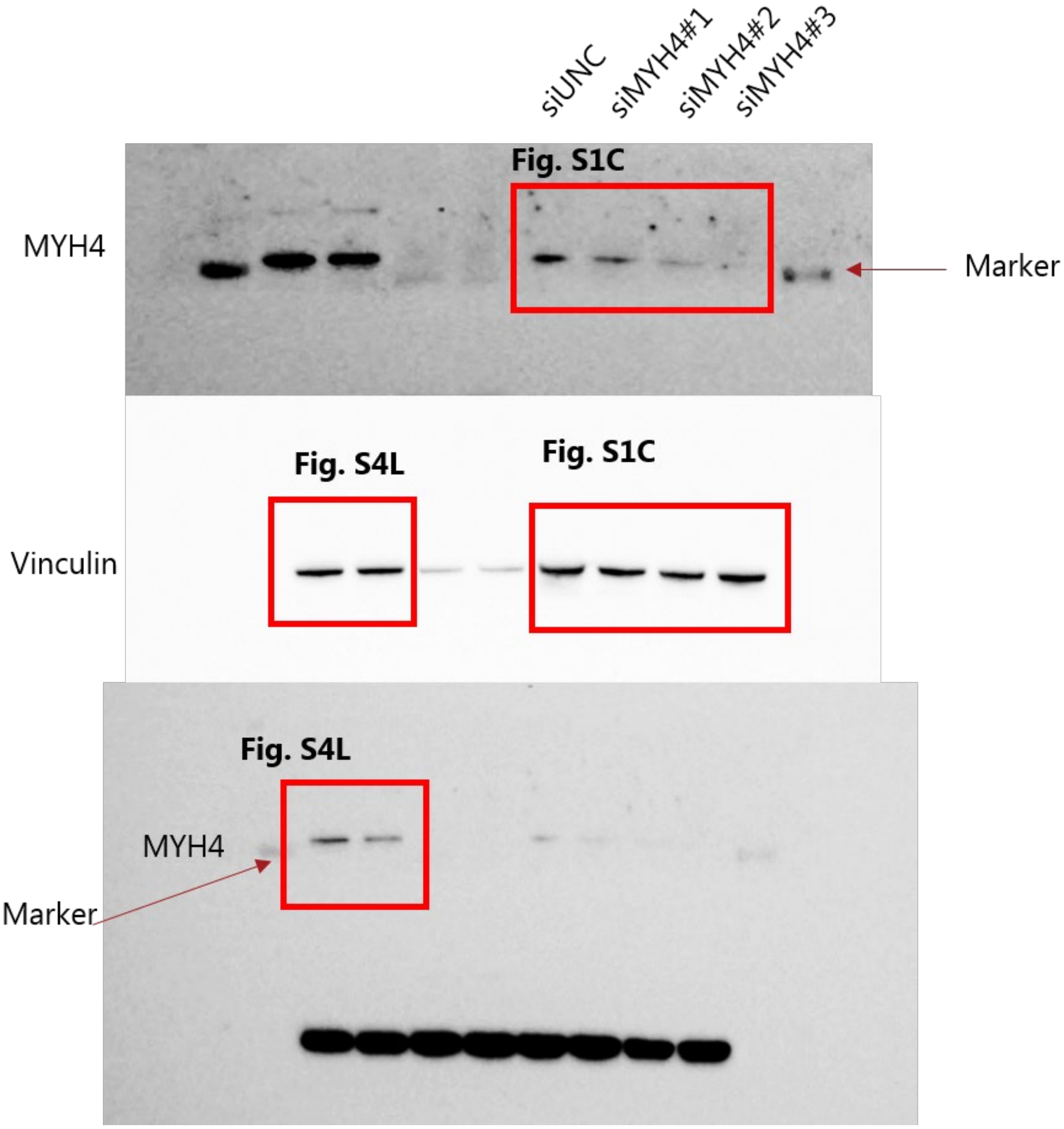

Fig. S1D

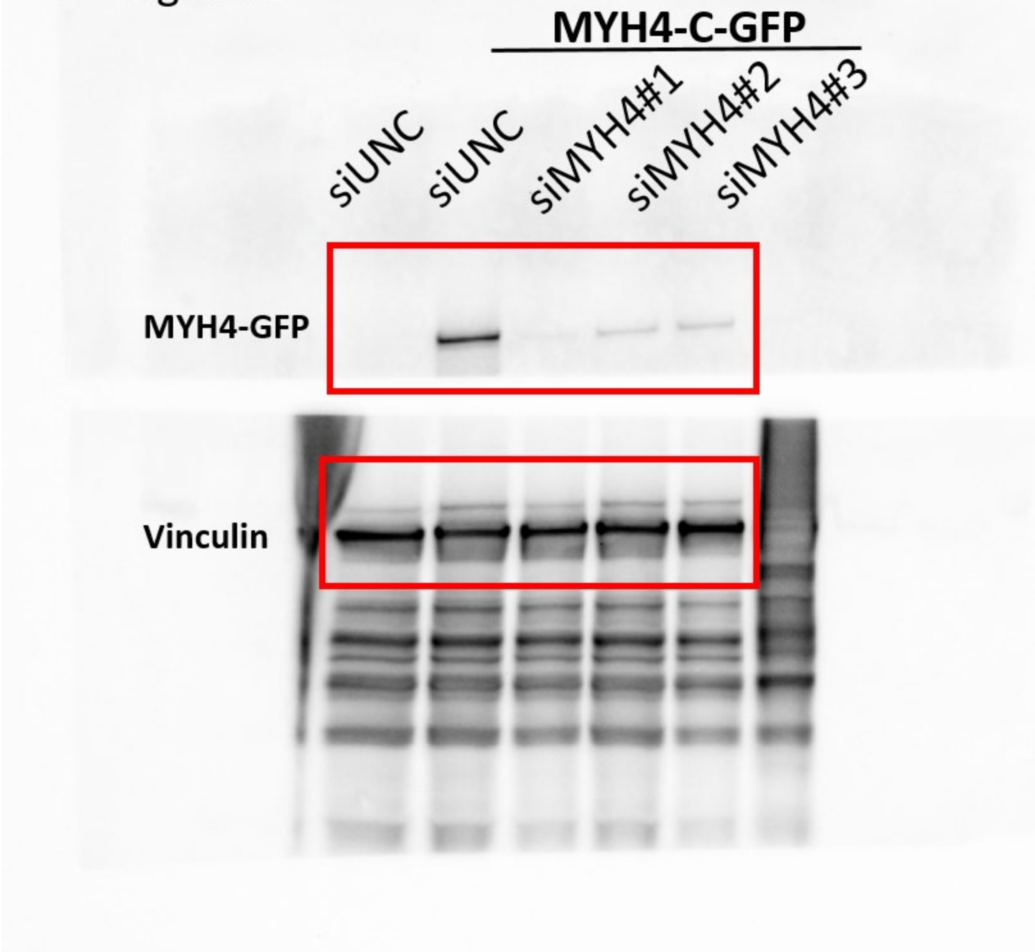
MYH4-C-GFP
siUNC
siUNC
siMYH4#1
siMYH4#2
siMYH4#3
MYH4-GFP
Vinculin

Fig. S2A

MYH4-GFP

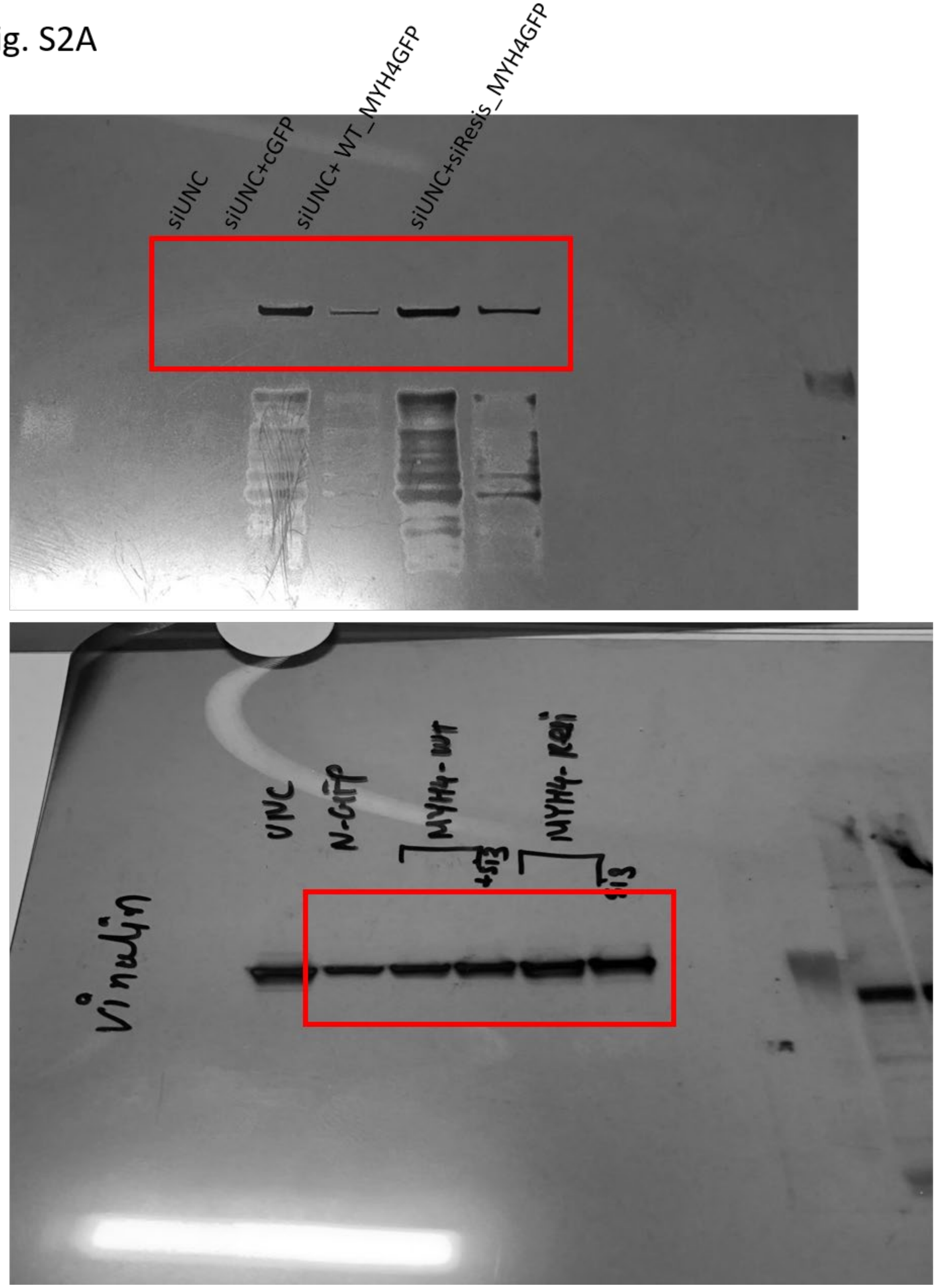

Vinculin

Fig. 3E panels

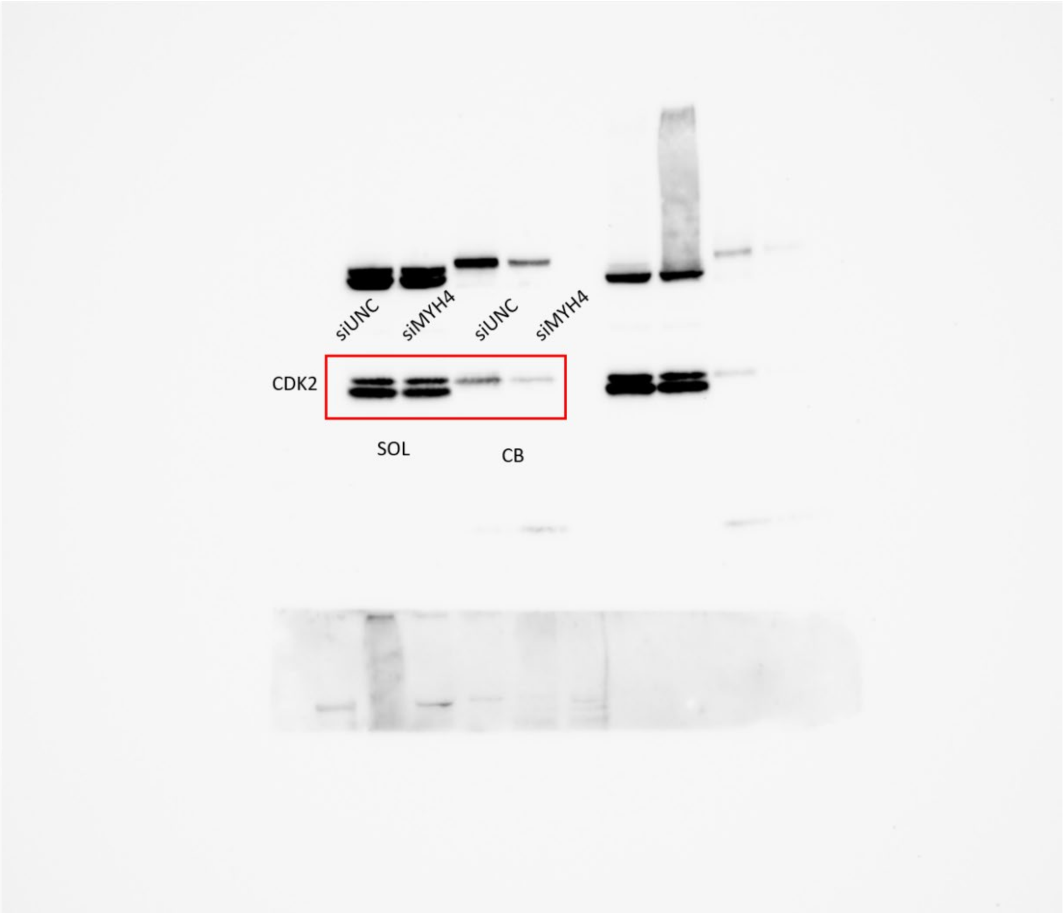

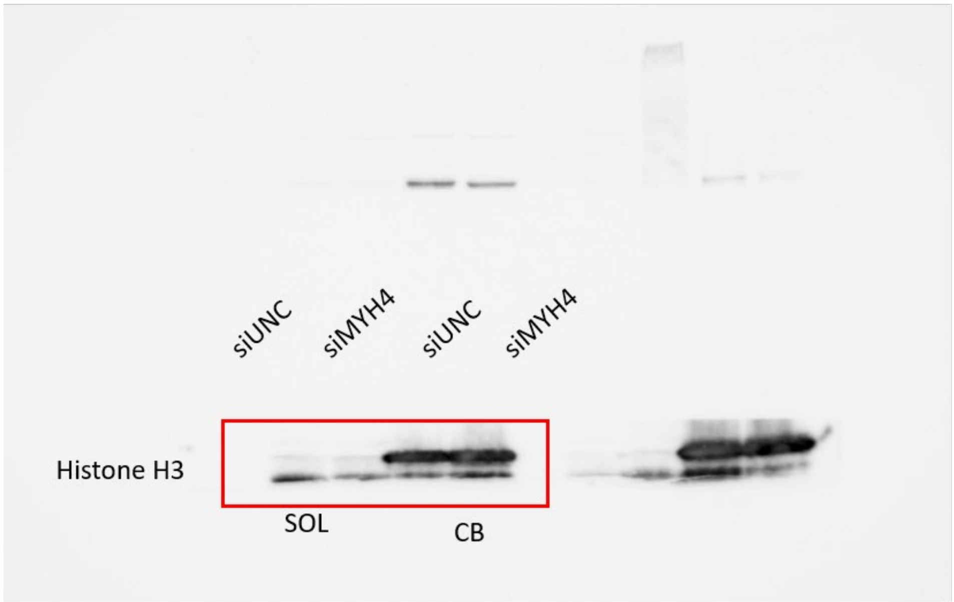
siUNC
siMYH4
siUNC
siMYH4
Histone H3
SOL
CB

18

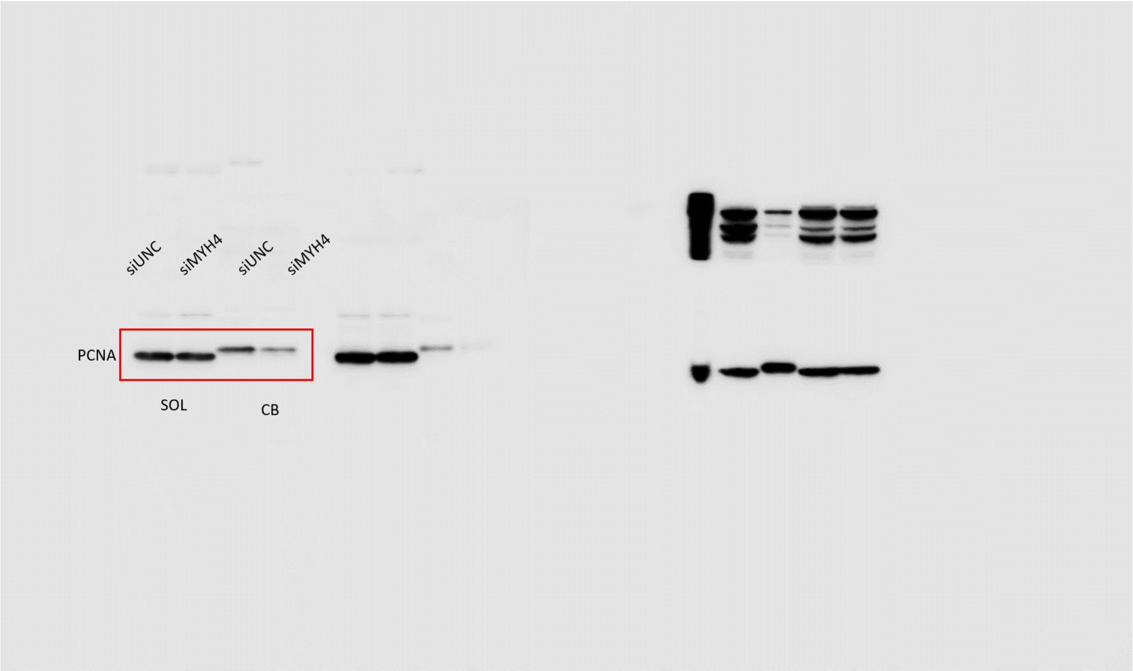
siUNC
siMYH4
siUNC
siMYH4
PCNA
SOL
CB

19

20

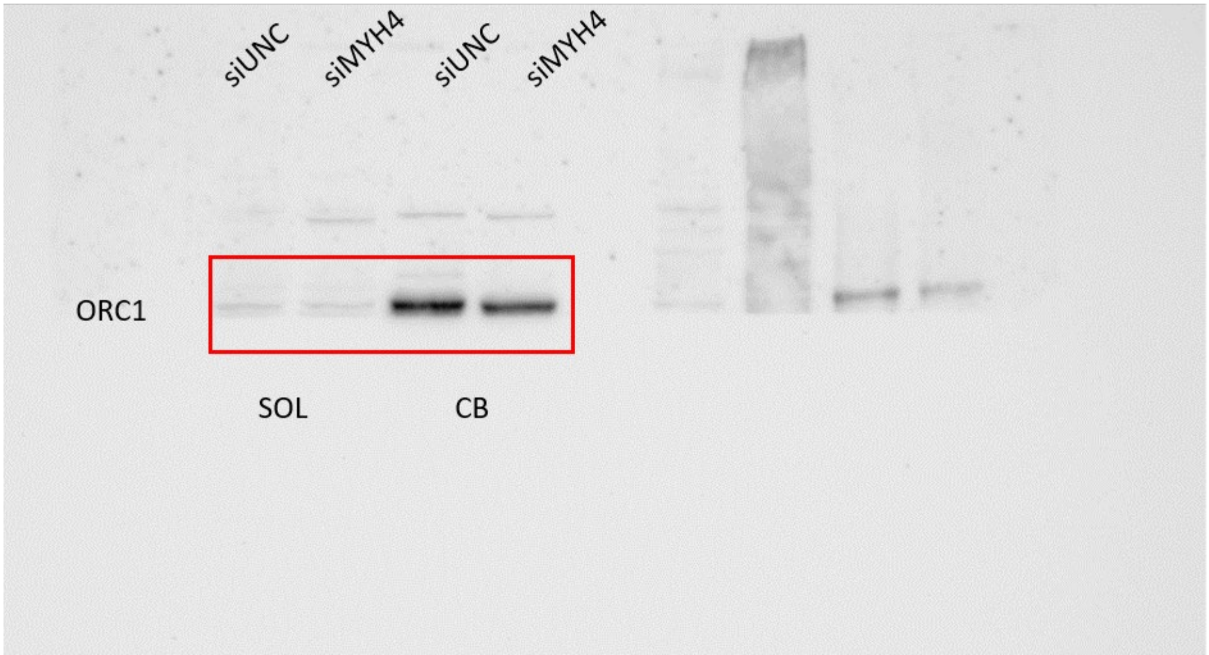

siUNC
siMYH4
siUNC
siMYH4
ORC1
SOL
CB

21

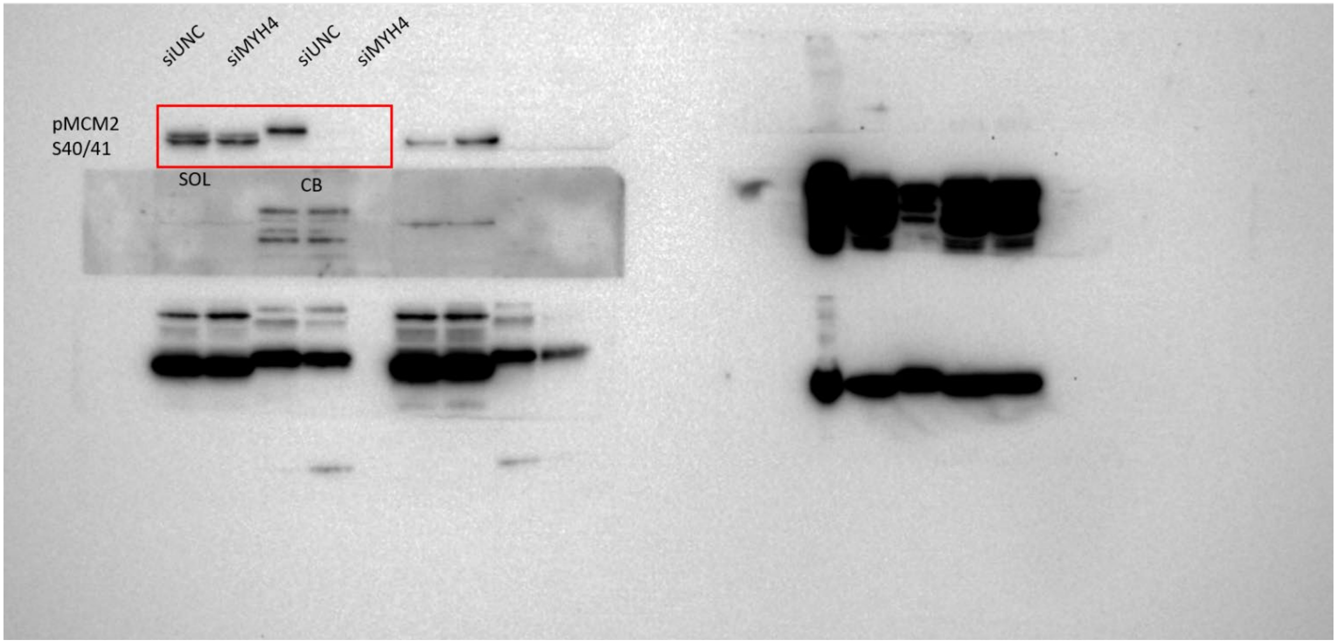

siUNC
siMYH4
siUNC
siMYH4
pMCM2
S40/41
SOL
CB

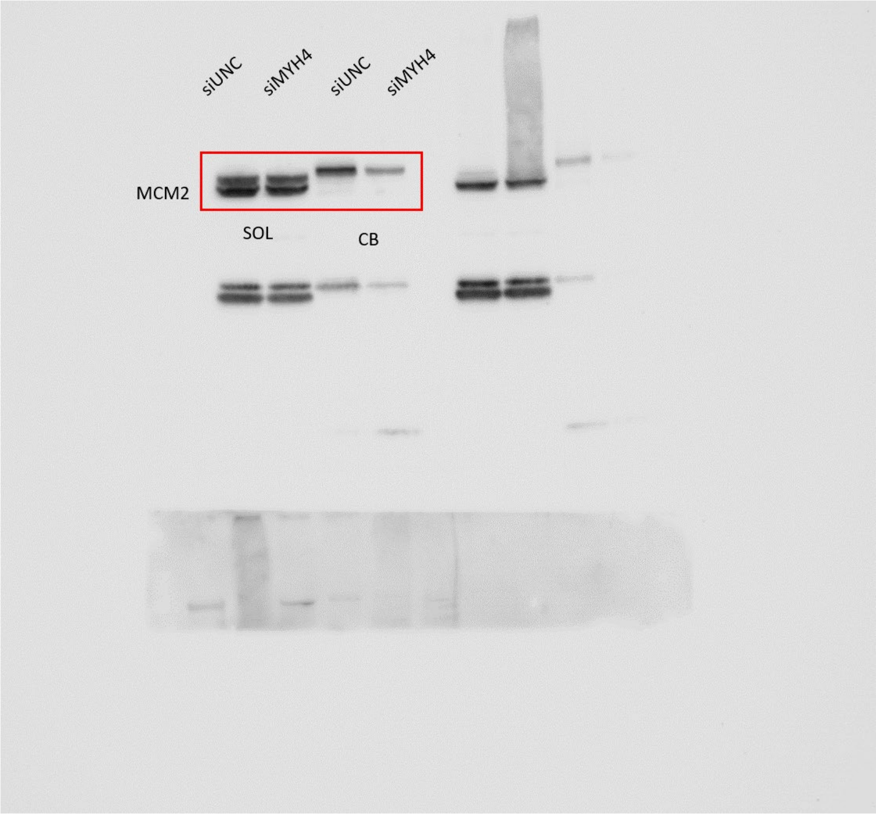
siUNC
siMYH4
siUNC
siMYH4
MCM2
SOL
CB

22

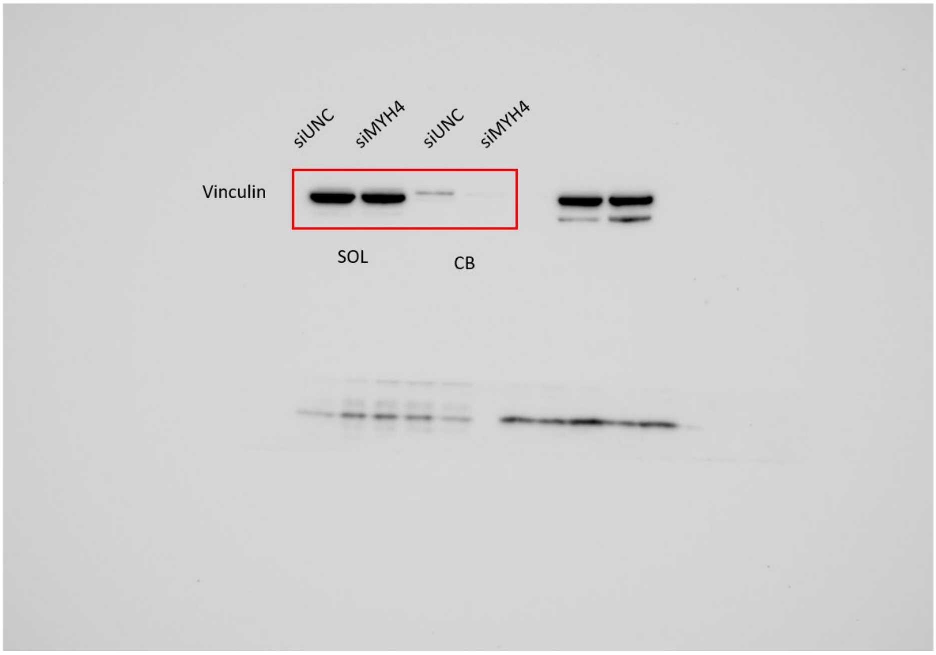
siUNC
siMYH4
siUNC
siMYH4
Vinculin
SOL
CB

23

Fig.S3E

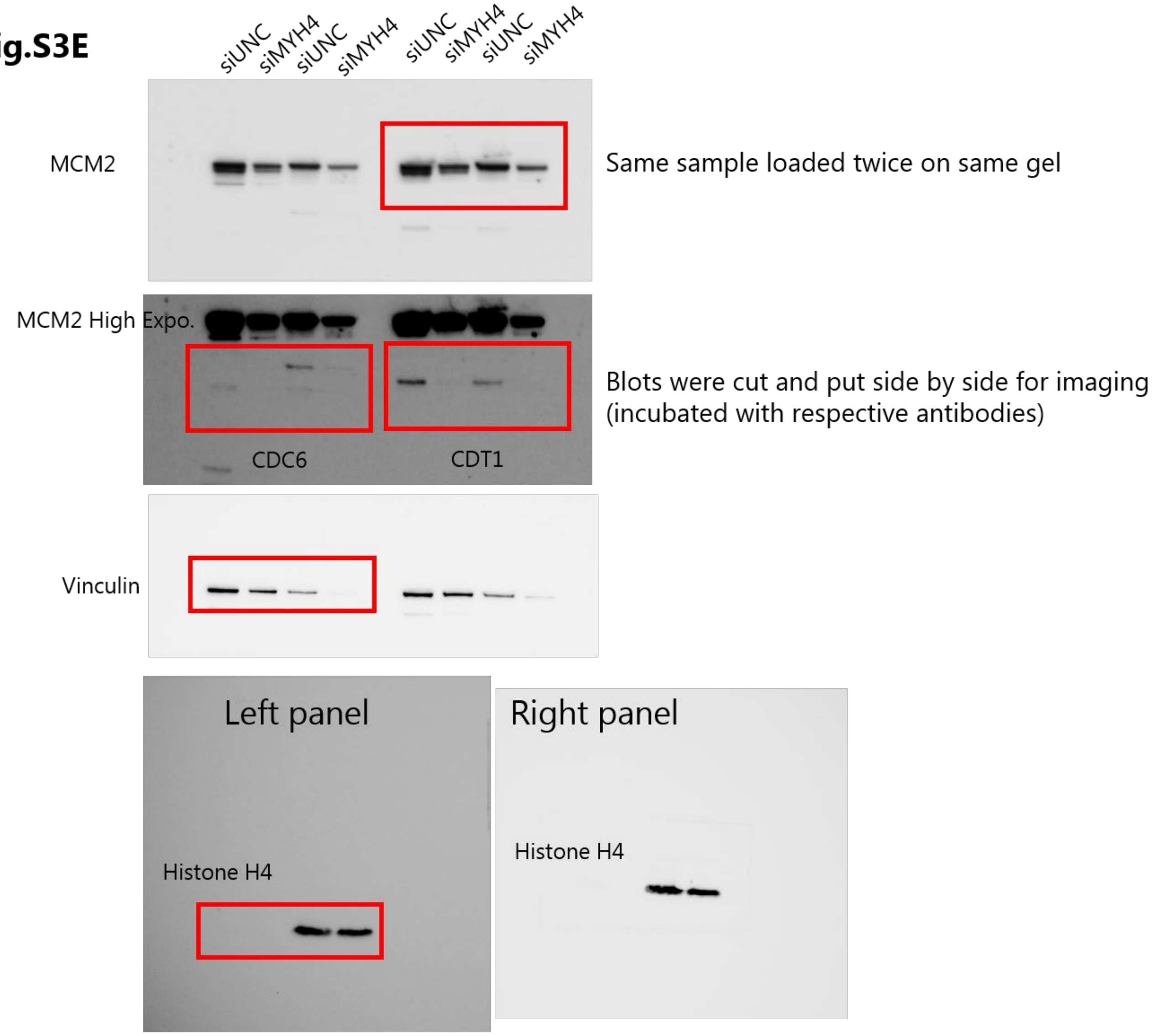
siUNC
siMYH4
siUNC
siMYH4
siUNC
siMYH4
siUNC
siMYH4
MCM2
Same sample loaded twice on same gel
MCM2 High Expo.
Blots were cut and put side by side for imaging (incubated with respective antibodies)
CDC6
CDT1
Vinculin
Left panel
Histone H4
Right panel
Histone H4
